# Incorporating deep and shallow components of genetic structure into the management of Alaskan red king crab

**DOI:** 10.1111/j.1752-4571.2012.00260.x

**Published:** 2012-12

**Authors:** William Stewart Grant, Wei Cheng

**Affiliations:** 1 Commercial Fisheries Division, Alaska Department of Fish and Game333 Raspberry Road, Anchorage, AK, USA; 2 Department of Biological Sciences, University of Alaska AnchorageAnchorage, AK, USA

**Keywords:** Bering Sea, genetic population structure, marine crustacean, mitochondrial DNA, northeastern Pacific, *Paralithodes camtschaticus*, phylogeography, single nucleotide polymorphisms

## Abstract

Observed patterns of genetic variability among marine populations are shaped not only by contemporary levels of gene flow, but also by divergences during historical isolations. We examined variability at 15 SNP loci and in mtDNA sequences (COI, 665 bp) in red king crab from 17 localities in the North Pacific. These markers define three geographically distinct evolutionary lineages (SNPs, *F*_CT_ = 0.054; mtDNA Φ_CT_ = 0.222): (i) Okhotsk Sea–Norton Sound–Aleutian Islands, (ii) southeastern Bering Sea–western Gulf of Alaska, and (iii) Southeast Alaska. Populations in the Bering Sea and in Southeast Alaska are genetically heterogeneous, but populations in the center of the range are homogeneous. Mitochondrial DNA diversity drops from *h* = 0.91 in the northwestern Pacific to *h* = 0.24 in the Southeast Alaska. Bayesian skyline plots (BSPs) indicate postglacial population expansions, presumably from ice-age refugia. BSPs of sequences simulated under a demographic model defined by late Pleistocene temperatures failed to detect demographic variability before the last glacial maximum. These results sound a note of caution for the interpretation of BSPs. Population fragmentation in the Bering Sea and in Southeast Alaskan waters requires population management on a small geographic scale, and deep evolutionary partitions between the three geographic groups mandate regional conservation measures.

## Introduction

Evolutionary biologists and marine fishery managers are challenged with two questions. First, how do genetic differences arise among marine populations? A prevailing paradigm postulates that populations diverge, in part, because of contemporary processes, including random drift and selection. Despite large potentials for dispersal of planktonic larvae in many marine species, shoreline topography and ocean currents, in some cases, isolate populations and promote divergence (Cowen and Sponaugle 2009). Ideally, the spatial scale of management should coincide with these natural subdivisions (Waples and Gaggiotti 2006). Second, how have historical events influenced the distribution of genetic diversity in contemporary populations? The legacies of historical events, such as ice-age vicariances, extinctions, dispersals, and colonizations, are not always considered when applying genetic data to management problems. Molecular markers can be used to address these questions by defining population boundaries and by placing the origins of population structures into a historical context. However, the application of genetic data to ecologically based management is not always straightforward, because genetic and ecological processes operate on different time scales (Waples et al. 2008; but see Watts et al. 2007).

**Figure 1:**
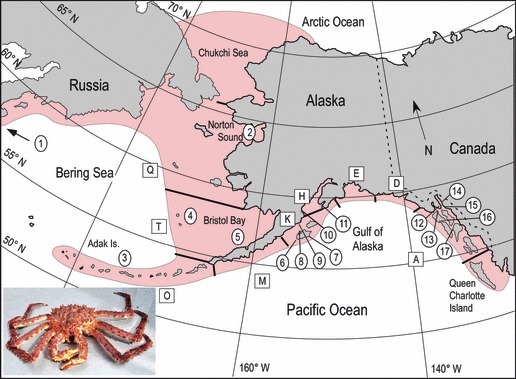
Map of the Bering Sea and Gulf of Alaska showing the geographic distribution of red king crab (red) and locations of samples. Numbers in ovals represent sample locations, and letters in squares represent State of Alaska harvest management areas (registration areas). Thick lines indicate management area boundaries.

Here, we focus on populations of *Paralithodes camtschaticus* (Tilesius, 1815; red king crab) in the North Pacific and Bering Sea. This species is the largest and most abundant of the king crabs in the North Pacific and extends from the Sea of Japan (Sato 1958) to northern British Columbia, Canada (Butler and Hart 1962), and into the Bering and Chukchi seas (Feder et al. 2005). In Alaska, harvests are managed on the basis of nine registration areas (Fig. 1: A, D, E, H, K, M, O, T, and Q). Additionally, the transport and release of invertebrates in Alaskan waters are regulated by six larval drift zones (based on ocean current patterns), which largely coincide with registration areas, except in the western Gulf of Alaska, which encompasses more than one registration area. Large fisheries in the southeastern Bering Sea and Gulf of Alaska have been reduced or closed (Otto 1986; Orensanz et al. 1998; Bechtol and Kruse 2009), because populations have declined owing to ocean–climate regime shifts (Mantua and Hare 2002; Kruse 2007) and overharvesting (Dew and McConnaughey 2005; Zheng and Kruse 2006; Bechtol and Kruse 2009). Declining harvests have prompted a consortium of stakeholders to develop hatchery technology to rebuild stocks through the large-scale release of hatchery stock (AKCRRAB 2011).

Several factors potentially influence genetic population structure of red king crab on historical and contemporary time scales. Adults move seasonally between deep water to feed and shallow water to breed (Dew 1990; Stone et al. 1992). Larval dispersal is initially limited, because developing eggs are attached to female pleopods for nearly a year before hatching (Marukawa 1933). After hatching, larvae drift in the fast-moving currents along Alaska’s coast (Shirley and Shirley 1989a) and influence population structure by promoting gene flow between populations (Bradbury et al. 2008; Selkoe and Toonen 2011). However, ocean fronts, mesoscale eddies (Hermann et al. 2002), complex shore lines, and habitat patchiness may limit larval movement.

On large temporal scales, climate cycles of cooling and warming greatly impacted the abundances and distributions of marine populations around the North Pacific (Grant and Utter 1984; Canino et al. 2010). Coastal tidewater glaciers covered much of the present range of red king crab in the northeastern Pacific (Mann and Hamilton 1995; Barrie and Conway 1999) and would have greatly reduced red king crab populations, because the completion of their life-history cycle depends on shallow-water and intertidal nursery areas (Dew 1990; Pirtle and Stoner 2010). Populations may have survived in refugia, particularly around Kodiak (Karlstrom and Ball 1969) and Queen Charlotte (Warner et al. 1982) islands, and along the southern shores of an unglaciated Bering Land Bridge (Hopkins 1972).

Previous genetic studies have partially resolved the genetic population structure of red king crab. An allozyme survey of populations in the eastern Bering Sea and Gulf of Alaska attempted to define population groups for harvest management and fishery enforcement (Seeb et al. 1990; Grant et al. 2011). These results, and others in Russian waters (Balakirev and Fedoseev 2000), showed that red king crab has low levels of allozyme variability, which may be due to historical population bottlenecks (Hall and Thatje 2009) or to strong metapopulation dynamics (Hedrick and Gilpin 1997). Other molecular markers have proved to be more polymorphic. Microsatellite loci, in particular, have high levels of polymorphism and have demonstrated small, but significant, allele-frequency differences between some populations. For example, Zelenina et al. (2008) found differences between Barents Sea populations founded in the 1960s, for which only low levels of divergence would be expected. Despite these differences, little heterogeneity existed between Barents Sea populations and Northwest Pacific donor populations. In the Northwest Pacific, no significant divergence was apparent between populations in the Sea of Japan and the eastern Okhotsk Sea. Another study of microsatellite variability showed no reduced genetic diversity in the Barents Sea populations (Jørstad et al. 2007).

The goal of this study was to use single nucleotide polymorphic (SNP) and mitochondrial (mt) DNA markers to define population structure for harvest management of red king crabs and to lay a foundation for possible stock enhancements. We use the analysis of allele frequencies to detect the effects of drift between populations, and the analysis of mtDNA variation to resolve historical events. Maternal inheritance of mtDNA produces lineages that trace the dispersals and colonizations that have shaped a deep phylogeographic structure in red king crabs (Avise 2000). We used molecular-clock methods to provide a temporal framework for reconstructions of population history (Arbogast et al. 2002). Our results show that both historical events and contemporary ocean-climate processes have produced an ocean-wide diversity gradient and a subdivided population structure in red king crabs that is unusual for a large crustacean.

## Materials and methods

### Samples collection and DNA extraction

In total, 1501 red king crabs were sampled at 17 locations extending from the Okhotsk Sea, through the Bering Sea and western Gulf of Alaska to Southeast Alaska (Fig. 1, Table 1). Crabs were collected by trawling, or by pots, from seven of nine Alaska harvest registration areas. Samples were taken during stock assessment surveys by NOAA Fisheries and the Alaska Department of Fish and Game (ADFG), and during test fisheries and commercial harvests. Some samples were used to survey allozyme variability among populations in Alaskan waters (Seeb et al. 1990; Grant et al. 2011). Small samples of crabs in some areas, or from successive years at the same location, were pooled to increase sample size. Samples from Deadman Reach (Southeast Alaska), collected in 1989 and 2001, were used to measure temporal heterogeneity in allele frequencies. In the present study, most of the same individuals were genotyped for SNPs and mtDNA. DNA was extracted from tissue samples using phenol–chloroform (Sambrook et al. 1989), DNeasy blood & tissue kits (QIAGEN, Valencia, CA, USA), or other inorganic methods.

**Table 1:** Summary statistics for single nucleotide polymorphisms (SNPs) and mitochondrial DNA (mtDNA) variability in samples of red king crab from 17 localities in the North Pacific

					SNPs						mtDNA						
							
	Sampling location	Date	N Latitude	Longitude	*N*	*H*_O_	*H*_E_	*F*_IS_	*P*	*N*	*N*_H_	*N*_R_	*h*	SD	Θ_π_	SD	*D*_T_
1	Okhotsk Sea, Russia	1998	53 30	155 38	51.0	0.180	0.201	0.104	0.048	46	21	19.0	0.911	0.025	0.0084	0.0046	−0.178
2	Norton Sound	2002	64 00	165 28	88.5	0.207	0.231	0.104	0.011	83	22	17.0	0.911	0.015	0.0086	0.0046	0.290
3	Adak Island	1988	53 00	175 34	80.3	0.202	0.226	0.107	0.002	83	15	10.8	0.833	0.027	0.0080	0.0043	0.982
4	Pribilof Islands	1996	56 51–57 25	169 35–170 31	91.1	0.220	0.223	0.016	–	95	14	10.4	0.729	0.042	0.0061	0.0034	−0.032
5	Bristol Bay	2001	57 45	159 00	78.6	0.193	0.208	0.073	–	51	13	11.5	0.751	0.052	0.0052	0.0030	−0.716
6	Alitak Bay, Kodiak Island	1988	56 47	154 16	84.9	0.201	0.206	0.023	–	94	11	8.8	0.612	0.055	0.0051	0.0029	−0.121
7	Chiniak Bay, Kodiak Island	1991	57 42	152 25	92.7	0.213	0.206	−0.032	–	82	9	7.2	0.556	0.059	0.0046	0.0027	−0.277
8	Uganik Passage, Kodiak Island	1991	57 38	153 13	91.3	0.188	0.206	0.087	0.013	94	10	8.2	0.589	0.057	0.0054	0.0030	0.204
9	Kukak Bay, Shelikof Strait	1991	58 18	154 16	60.8	0.148	0.152	0.028	0.001	61	9	8.2	0.594	0.069	0.0047	0.0028	−0.235
10	Kamishak Bay, Cook Inlet	2001	59 12	153 45	61.9	0.210	0.218	0.038	–	29	9	9.0	0.741	0.075	0.0067	0.0038	0.322
11	Kachemak Bay, Cook Inlet	1988	59 42	151 11	42.9	0.250	0.220	−0.138	0.002	43	7	6.8	0.639	0.075	0.0063	0.0035	0.663
12	St. James Bay	1988	58 05	135 12	89.6	0.208	0.213	0.027	–	94	7	4.8	0.219	0.057	0.0004	0.0005	−1.787^*^^*^
13	Eagle River	1988	58 30	143 52	92.3	0.193	0.204	0.053	–	66	5	3.8	0.118	0.054	0.0002	0.0003	−1.833^*^^*^
14	Barlow Cove	1991	58 20	134 53	87.9	0.187	0.205	0.090	0.022	65	7	5.5	0.231	0.069	0.0005	0.0006	−1.903^*^^*^
15	Seymour Canal	2001	57 49	134 01	46.9	0.202	0.208	0.030	–	40	5	4.8	0.314	0.091	0.0005	0.0006	−1.538^*^
16	Deadman Reach	1989	57 31	135 30	92.5	0.209	0.215	0.028		90	9	6.5	0.304	0.063	0.0005	0.0006	–
	Deadman Reach	2001	57 31	135 30	90.2	0.191	0.198	0.035		90	7	5.2	0.229	0.059	0.0004	0.0005	–
	Pooled				182.7	0.200	0.206	0.030		180	10	5.9	0.266	0.044	0.0004	0.0005	−1.962^*^^*^
17	Gambier Bay	1988–1989	57 27	133 57	93.7	0.204	0.205	0.005		69	10	7.2	0.27	0.071	0.0004	0.0005	−2.237^*^^*^^*^

SNPs: average sample size over 15 loci, observed (*H*_O_) and expected (*H*_E_) heterozygosity for 15 single nucleotide polymorphisms, inbreeding coefficient (*F*_IS_), probability of departure from Hardy–Weinberg proportions over loci (*P*). mtDNA: average sample size among SNP loci or sample size for mtDNA (N), number of haplotypes (*N*_H_), haplotype richness (*N*_R_), haplotype diversity (*h*), nucleotide diversity (θ_π_), Tajima’s *D* (*D*_T_), ^*^P < 0.05, ^*^^*^P < 0.01, ^*^^*^^*^P < 0.001

### Nuclear single nucleotide polymorphism genotyping

Fifteen nuclear SNP loci (An et al. 2010; Table S1) were genotyped in 384-well reaction plates. Each 5-μL reaction mixture consisted of 1.0 μL template DNA in 1× TaqMan Universal Buffer from Applied Biosystems Inc. (ABI, Foster City, CA, USA), 900 nm of both polymerase chain reaction (PCR) primers, and 200 nm of each probe (Table S1). SNP amplifications were conducted with an ABI 9700 thermocycler with an initial denaturation of 10 min at 95°C, 50 cycles of 1 s at 92°C, and 1 min at primer-specific annealing temperatures 58, 59, or 60°C (Table S1). SNP amplifications were made with a ramp speed of 1°C/s. After amplification, plates were read and scored with an ABI 7900 HT real-time PCR instrument using SEQUENCE DETECTION 2.2 (ABI).

### Mitochondrial DNA sequencing

The mitochondrial DNA cytochrome oxidase subunit I (COI) universal primers LCO1490 (5′-GGTCAACAAATCATAAAGATATTGG-3′) and HCO2198 (5′-TAAACTTCAGGGTGACCAAAAAATCA-3′; Folmer et al. 1994) were used to amplify a 665-bp fragment (‘barcode’ segment). PCR mixtures consisted of a 50-μL mixture of 2.0 μL template DNA in 1× Colorless GoTaq Flexi Buffer, 2.5 mm MgCl_2_, 1 mm dNTPs (ABI), 1 μm of forward and reverse primers, and 5 U GoTaq Flexi DNA polymerase (Promega Inc., Madison, WI, USA). PCR amplifications were conducted in ABI 9700 thermocyclers with an initial denaturation of 1 min at 95°C, 37 cycles of 40 s at 95°C, 40 s at primer annealing temperature 41°C, and 1 min at 72°C; the final cycle was at 4°C for 5 min. Cycles were made at a ramp speed of 1°C/s. The PCR amplifications were sequenced in the forward and reverse directions by the High-Throughput Genomics Unit (Seattle, WA, USA). Sequences were aligned with a published sequence for red king crab (GenBank accession no. AB211303) with the muscle algorithm (mega 5, Tamura et al. 2011) and adjusted by eye. Polymorphic sites were confirmed with reverse sequences. GenBank accession nos. (JF738153–JF738249) also include sequences shared with the study of Zelenina et al. (2008).

### Statistical analyses

Single nucleotide polymorphism genotypes for the 15 loci were tested for fit of genotypic frequencies to Hardy–Weinberg expectations (HWE) in each sample with exact probability tests of 20 batches of 10 000 permutations and a burn-in of 10 000 permutations (genepop 4, Rousset 2008). Probabilities over loci per sample, over loci, and over samples were determined with Fisher’s method. Sample heterozygosities were estimated with genepop. Linkage disequilibrium between loci in each sample and overall was tested with genepop with 100 batches of 5000 iterations and with a burn-in of 10 000 iterations. Analysis of molecular variation (amova) in arlequin 3.11 (Excoffier et al. 2005) was used to explore geographic and temporal structures in both SNP allele frequencies and mtDNA haplotypes. These amovas were based on *F*_ST_ estimated from allele (SNPs) or haplotype (mtDNA) frequencies. Additional amovas of mtDNA haplotypes were based on an *F*_ST_ analog statistic (Φ_ST_) that accounts for both haplotype-frequency differences between samples and sequence divergences between haplotypes. We used the Tamura and Nei (1993) substitution model in the amovas. The probability an *F* statistic was larger than 0.0 was estimated with 50 000 permutations of individuals among samples.

Statistical power to detect significant differences with 15 SNP loci and 17 samples was estimated with powsim 4.0 (Ryman and Palm 2006), using *N*_e_ = 2000 and 1000 replicate runs. The incorporation of divergences between haplotypes in tests of differentiation with amova and estimates of Φ_ST_ adds additional power to tests of geographic structure. Geographic groupings of samples were visualized with principal coordinates analysis (PCoA) computed with NTSYS (Exeter Software; Exeter, New York, NY, USA). Haplotype richness was estimated with HP-rare (Kalinowski 2004, 2005) with a minimal sample size of 29 crabs. Mitochondrial DNA nucleotide diversity (Θ_π_) and haplotype diversity (*h*) and their standard deviations were estimated with arlequin. A 95% plausible parsimony network of mtDNA haplotypes in 1278 crabs was made with TCS 1.21 (Clement et al. 2002). This network was used as a template to construct networks for the three regional groups identified with amova.

Historical demographies were examined in two ways. First, we tested for population and spatial range expansions with mismatch distributions between haplotypes using arlequin. Both recent demographic and spatial expansions are expected to produce a unimodal mismatch distribution from the accumulation of mutations (Ray et al. 2003). With time, the mode of the distribution increases and gives way to a multimodal mismatch distribution (Rogers and Harpending 1992). The time since the population expansion can be estimated with τ = 2*ut*, where τ is estimated from the distribution, *u* is the whole-sequence mutation rate and *t* is time since expansion. The use of mismatch distributions is discussed further in Table S7. Tajima’s *D*_T_ was estimated from the distributions of haplotype frequencies in a sample and used to test for neutrality (Tajima 1989). Significance was determined with 10 000 randomizations in arlequin. Population expansions can lead to excesses of low-frequency haplotypes before populations reach drift–mutation equilibria.

Historical demographies of the three major groups were examined with coalescence theory and Bayesian skyline plots (BSPs) with beast 1.6.1 (Drummond and Rambaut 2007) with MCMC runs of up to 400 million steps that yielded effective sample sizes (ESS) of at least 200. Effective population size (*N*_e_) can be estimated from the results with *N*_e_μ*g*, where μ is the mutation rate and *g* is generation time (about 5 years for red king crab). However, estimates of μ are problematic, as no temporally anchored phylogenetic estimates are available for *Paralithodes*. Even so, phylogenetic estimates appear to greatly underestimate contemporary mutation rates and do not appear to be appropriate for BSP calibrations (Ho et al. 2011). To help interpret the results of the BSPs, we simulated samples (*n* = 200) of sequences (*bp* = 665) with Mesquite 2.74 (Maddison and Maddison 2010). The scaling factor (roughly mutation rate) and base effective population size were varied until the simulated sequences had similar haplotype and nucleotide diversities (within ± SE) as those in the three major groups of red king crab. We used a demographic model based on Antarctic ice core paleoclimatic temperatures over the last four glacial cycles reaching to 450 kyr (Jouzel et al. 2007). We reasoned that abundances of red king crabs were proportional to the amount of shallow-water habitat, which was related exponentially to a linear increment in warming following the LGM. BSPs were constructed from the sequences with beast and with appropriate substitution models, as determined with jModelTest (Posada 2008). This empirical approach to calibrating the molecular clock is similar to that described by Crandall et al. (2012), who used the post-Pleistocene expansion onto the Sunda Shelf to date a genetic signature of population growth in three invertebrates.

## Results

### SNP variability

**Figure 2:**
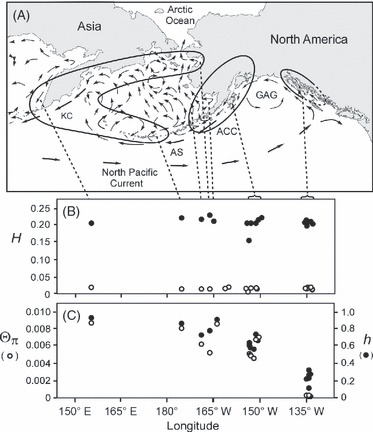
(a) Map of the North Pacific Ocean and Bering Sea showing generalized current patterns and three major population groups of red king crabs. (b) Average heterozygosity of 15 single nucleotide polymorphisms (closed circles) and average heterozygosity of 38 allozyme loci (open circles). (c) Mitochondrial DNA haplotype diversity (closed circles) and nucleotide diversity (open circles). KC, Kamchatka Current; AS, Alaska Stream; ACC, Alaska Coastal Current; and GAG, Gulf of Alaska Gyre.

Fourteen of 15 SNP loci had common-allele frequencies <0.95 in at least one of the 18 samples examined (Table S2). Observed heterozygosities varied from *H*_O_ = 0.148 (western Gulf of Alaska) to *H*_O_ = 0.220 (Pribilof Islands; Table 1). Seven significant departures from Hardy–Weinberg proportions appeared among samples: six departures reflected heterozygote deficits, and one a heterozygote excess. None of the tests for linkage disequilibrium between loci within samples, or over all samples, were significant after Bonferonni adjustment of rejection probabilities. Expected heterozygosities ranged from *H* = 0.152 to 0.231 among samples and did not show a geographic trend (Table 1, Fig. 2b).

Over all the 17 samples, powsim indicated that the probability of a type I error was α = 0.05, and the chance of detecting a significant difference of *F*_ST_ = 0.001 was 0.524, and of *F*_ST_ = 0.01 was 1.0. However, large breaks occurred between population groups, and we were interested in divergences between populations within these groups. The probability of detecting a significant divergence for *F*_ST_ = 0.001 among samples 1, 2, and 3 in the Bering and Okhotsk seas was 0.123, and 0.950 for *F*_ST_ = 0.01. In the western Gulf of Alaska and southeastern Bering Sea, the probability of detecting a significant *F*_ST_ = 0.001 among samples 4–11 was 0.255, and 1.0 for *F*_ST_ = 0.01. Among southeast Alaska populations, power for detecting a significant *F*_ST_ = 0.001 was 0.261, and 1.0 for *F*_ST_ = 0.01.

**Figure 3:**
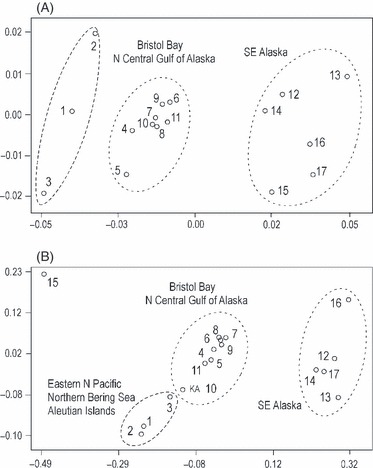
Two-dimensional principal coordinate plot of red king crab (a) SNPs and (b) mtDNA variability among populations in the North Pacific and Bering Sea.

Values of *F*_ST_ between samples ranged from 0.0 to 0.091 (Table S3). A PCoA of these *F*_ST_ values resolved three groups of samples (Fig. 3a). Samples 1, 2, and 3 were marginally divergent from one another, but strongly divergent from the other samples. Samples from the southeastern Bering Sea [4, 5] and western Gulf of Alaska [6–11] comprised a second group, and samples from Southeast Alaska [12–17] comprised a third group. Consistent with the amovas, samples from SE Alaska tended to show a greater amount of genetic separation than did the samples in the southeastern Bering Sea and western Gulf of Alaska group.

No significant temporal shift in SNP frequencies overall loci was detected between two samples from Deadman Reach (1989 and 2001; *F*_SC_ = −0.009, *P* = 0.999, df = 14), so these samples were combined. SNP allele frequencies differed significantly among the 17 samples (*F*_SC_ = 0.041, *P* < 0.00001, df = 224). An amova indicated that the greatest heterogeneity occurred among four groups, [1] [2, 3] [4–11] [12–17] (*F*_CT_ = 0.054, *P* < 0.00001, df = 56), or among three groups [1–3] [4–11] [12–17] (*F*_CT_ = 0.054, *P* < 0.00001, df = 42; Table 2). Significant heterogeneity appeared among samples 1, 2, and 3 (*F*_SC_ = 0.031, *P* < 0.00001, df = 42). Samples 1 and 3 were significantly different (*F*_CT_ = 0.032, *P* = 0.001, df = 14), but not 1 and 2 (*F*_CT_ = 0.002, *P* = 0.335, df = 14). Eight samples from the southeastern Bering Sea and western Gulf of Alaska [4–11] were genetically homogeneous (*F*_SC_ = 0.002, *P* = 0.228, df = 112). However, a comparison between samples from the southeastern Bering Sea and western Gulf of Alaska was significant (*F*_CT_ = 0.004, *P* = 0.003, df = 14). A significant amount of heterogeneity appeared among the six samples from southeast Alaska [12–17] (*F*_SC_ = 0.007, *P* = 0.009, df = 70). A core group of samples [12, 13, 14, 17] showed no heterogeneity (*F*_SC_ = 0.004, *P* = 0.108, df = 42). However, samples 15 (*F*_SC_ = 0.001, *P* = 0.019, df = 56) and 16 (*F*_ST_ = 0.005, *P* = 0.033, df = 56) each introduced heterogeneity into this core group.

**Table 2:** Analysis of molecular variance (amova) of 15 single nucleotide polymorphisms (SNPs) in red king crab. Sample groupings for the various amovas are indicated by asterisks and brackets

Comparison																			amova			
	
1	2	3	4	5	6	7	8	9	10	11	12	13	14	15	16	16a	16b	17	*F*_CT_	*P*	*F*_SC_	*P*
–	–	–	–	–	–	–	–	–	–	–	–	–	–	–	–	[^*^	^*^]	–	–	–	−0.009	0.999
[^*^]	[^*^	^*^]	[^*^	^*^	^*^	^*^	^*^	^*^	^*^	^*^]	[^*^	^*^	^*^	^*^	^*^	–	–	^*^]	0.054	<0.00001	0.005	0.025
[^*^	^*^	^*^]	[^*^	^*^	^*^	^*^	^*^	^*^	^*^	^*^]	[^*^	^*^	^*^	^*^	^*^	–	–	^*^]	0.054	<0.00001	0.005	0.012
[^*^	^*^	^*^]	–	–	–	–	–	–	–	–	–	–	–	–	–	–	–	–	–	–	0.031	<0.00001
[^*^	^*^]	–	–	–	–	–	–	–	–	–	–	–	–	–	–	–	–	–	–	–	0.002	0.335
[^*^	–	^*^]	–	–	–	–	–	–	–	–	–	–	–	–	–	–	–	–	–	–	0.032	0.001
–	–	–	[^*^	^*^	^*^	^*^	^*^	^*^	^*^	^*^]	–	–	–	–	–	–	–	–	–	–	0.002	0.228
–	–	–	[^*^	^*^]	[^*^	^*^	^*^	^*^	^*^	^*^]	–	–	–	–	–	–	–	–	0.004	0.003	0.001	0.630
–	–	–	[^*^	^*^]	–	–	–	–	–	–	–	–	–	–	–	–	–	–	–	–	0.005	0.951
–	–	–	–	–	[^*^	^*^	^*^	^*^	^*^	^*^]	–	–	–	–	–	–	–	–	–	–	−0.003	0.930
–	–	–	–	–	–	–	–	–	–	–	[^*^	^*^	^*^	^*^	^*^	–	–	^*^]	–	–	0.007	0.009
–	–	–	–	–	–	–	–	–	–	–	[^*^	^*^]	–	–	–	–	–	–	–	–	0.003	0.267
–	–	–	–	–	–	–	–	–	–	–	[^*^	^*^	^*^]	–	–	–	–	–	–	–	0.003	0.201
–	–	–	–	–	–	–	–	–	–	–	[^*^	^*^	^*^	^*^]	–	–	–	–	–	–	0.009	0.022
–	–	–	–	–	–	–	–	–	–	–	[^*^	^*^	^*^	–	^*^]	–	–	–	–	–	0.007	0.017
–	–	–	–	–	–	–	–	–	–	–	[^*^	^*^	^*^	–	–	–	–	^*^]	–	–	0.004	0.108
–	–	–	–	–	–	–	–	–	–	–	[^*^	^*^	^*^	^*^	–	–	–	^*^]	–	–	0.001	0.019
–	–	–	–	–	–	–	–	–	–	–	[^*^	^*^	^*^	–	^*^	–	–	^*^]	–	–	0.005	0.033

Sample numbers as in Table 1. *F*_CT_ is differentiation among groups indicated by brackets, and *F*_SC_ is the average differentiation among samples within groups. *P* is the probability that *F*_CT_ and *F*_SC_ are significantly >0.0.

### Mitochondrial DNA sequence variability

**Figure 4:**
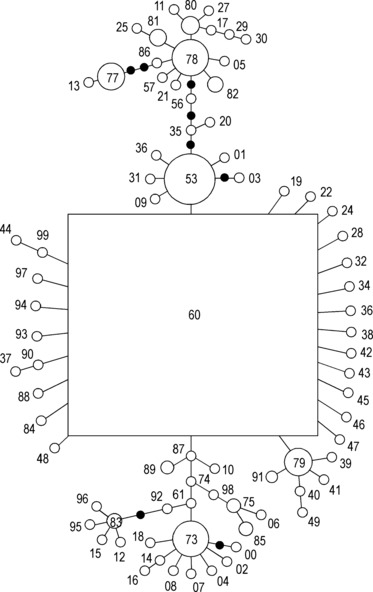
Parsimony haplotype network for the pooled sample of red king crabs (*n* = 1278) from the North Pacific. The central square represents a hypothesized ancestral haplotype. Open circles represent observed haplotypes, and closed circles represent hypothetical, unobserved haplotypes. Symbol size is proportion to haplotype frequency. Haplotype labels are the last two digits of haplotypes with GenBank accession numbers JF738153–JF38249.

A 665-bp segment of COI was sequenced in 1278 crabs from 17 localities with an average sample size of 70.8 crabs (range *N* = 21–94; Tables 1 and S4). Sixty-four substitutions (transitions = 51, transversion = 13) at 59 polymorphic nucleotide sites defined 81 haplotypes in a complex genealogy (Fig. 4). Haplotype richness varied clinally across the North Pacific, with the largest value *N*_R_ = 19.0–17.0 in western samples to *N*_R_ = 3.8–7.2 in samples from Southeast Alaska. A longitudinal cline also appeared in haplotype diversities, which shifted from *h* = 0.911 in western samples to *h* = 0.236 among Southeast Alaska samples [12–17] (Table 1, Fig. 2c). Mean nucleotide diversities dropped from Θ_π_ = 0.0085 in western samples to Θ_π_ = 0.0004 in Southeast Alaska samples.

powsim simulations indicated that the power to detect a divergence of *F*_ST_ = 0.001 with mtDNA haplotype frequencies was low, ranging from *P* = 0.110 to 0.275 for divergences among populations within the Bering Sea, western Gulf of Alaska, and Southeast Alaska. However, the power to detect a significant *F*_ST_ = 0.01 was >0.999 for these three groups.

Values of *F*_ST_ between samples ranged from 0.0 to 0.803 (Table S5), and values of Φ_ST_ ranged from 0.0 to 0.833 between samples (Table S6). As for SNPs, the two temporal samples from Deadman Reach (1989 and 2001) were not significantly different from one another (Table 3; *F*_ST_ = −0.005; *P* = 0.936; Φ_ST_ = −0.007, *P* = 0.937, df = 1) and were pooled. A PCoA of Φ_ST_ values revealed the same three groups detected with SNPs, with an addition of the outlier sample from Seymour Canal (Fig. 3b). Crabs in Seymour Canal [15] carried a unique lineage not present in other samples. In this PCoA, 1, 2, and 3 were not well separated. A central group included samples from the Southeastern Bering and the Gulf of Alaska. The last group included five samples from Southeast Alaska [12–14, 16, 17].

**Table 3:** Analysis of molecular variance (amova) of mitochondrial DNA (cytochrome oxidase subunit I) variability among populations of red king crab in the North Pacific. Sample groupings for the various amovas are indicated by asterisks and brackets

Comparison																			amova							
	
1	2	3	4	5	6	7	8	9	10	11	12	13	14	15	16	16a	16b	17	*F*_CT_	*P*	Φ_CT_	*P*	*F*_SC_	*P*	Φ_SC_	*P*
–	–	–	–	–	–	–	–	–	–	–	–	–	–	–	–	[^*^]	[^*^]	–	–	–	–	–	−0.005	0.936	−0.007	0.937
[^*^]	[^*^	^*^]	[^*^	^*^	^*^	^*^	^*^	^*^	^*^	^*^]	[^*^	^*^	^*^	^*^	^*^	–	–	^*^]	0.129	0.006	0.218	0.00006	0.129	<0.00001	0.074	<0.00001
[^*^	^*^	^*^]	[^*^	^*^	^*^	^*^	^*^	^*^	^*^	^*^]	[^*^	^*^	^*^	^*^	^*^	–	–	^*^]	0.138	0.002	0.222	0.00008	0.124	<0.00001	0.072	<0.00001
[^*^	^*^	^*^]	–	–	–	–	–	–	–	–	–	–	–	–	–	–	–	–	–	–	–	–	0.038	<0.00001	0.029	0.007
[^*^	^*^]	–	–	–	–	–	–	–	–	–	–	–	–	–	–	–	–	–	–	–	–	–	0.033	0.0007	0.034	0.027
[^*^	–	^*^]	–	–	–	–	–	–	–	–	–	–	–	–	–	–	–	–	–	–	–	–	0.006	0.197	0.001	0.332
–	–	–	[^*^	^*^	^*^	^*^	^*^	^*^	^*^	^*^]	–	–	–	–	–	–	–	–	–	–	–	–	0.005	0.095	−0.001	0.527
–	–	–	[^*^	^*^]	[^*^	^*^	^*^	^*^	^*^	^*^]	–	–	–	–	–	–	–	–	0.016	0.041	0.001	0.432	−0.002	0.581	−0.013	0.521
–	–	–	[^*^	^*^]	–	–	–	–	–	–	–	–	–	–	–	–	–	–	–	–	–	–	0.002	0.313	−0.004	0.532
–	–	–	–	–	[^*^	^*^	^*^	^*^	^*^	^*^]	–	–	–	–	–	–	–	–	–	–	–	–	−0.002	0.620	−0.00004	0.459
–	–	–	–	–	–	–	–	–	–	–	[^*^	^*^	^*^	^*^	^*^	–	–	^*^]	–	–	–	–	0.357	<0.00001	0.400	<0.00001
–	–	–	–	–	–	–	–	–	–	–	[^*^	^*^	^*^	^*^	–	–	–	^*^]	–	–	–	–	0.496	<0.00001	0.504	<0.00001
–	–	–	–	–	–	–	–	–	–	–	[^*^	^*^	^*^	–	^*^	–	–	^*^]	–	–	–	–	−0.001	0.519	0.001	0.261

Sample numbers as in Table 1. *F*_CT_ and Φ_CT_ are measures of differentiation among groups indicated by brackets, and *F*_SC_ and Φ_SC_ are the average differentiation among samples within groups. *P* is the probability that the statistics of differentiation are significantly >0.0.

A significant amount of overall haplotype-frequency heterogeneity (*F*_CT_ = 0.207, *P* < 0.00001, df = 16) and haplotype divergence (Φ_CT_ = 0.217, *P* < 0.00001, df = 16) corresponds to large differences among groups. Most of this divergence was because of differences among the three regional groups [1–3] [4–11] [12–17] (*F*_CT_ = 0.138, *P* = 0.002; Φ_CT_ = 0.222, *P* = 0.00008, df = 2) (Table 3). The comparison of samples 1, 2, and 3 was significant (*F*_CT_ = 0.038, *P* < 0.00001; Φ_CT_ = 0.029, *P* = 0.007, df = 2). Unlike SNPs, samples 1 and 2 differed significantly (*F*_SC_ = 0.033, *P* = 0.0007; Φ_SC_ = 0.034, *P* = 0.027, df = 1), but not 1 and 3 (*F*_SC_ = 0.006, *P* = 0.197; Φ_SC_ = 0.001, *P* = 0.332, df = 1). As with SNPs, samples 4 and 5 from the southeastern Bering Sea were not significantly different from each other (*F*_SC_ = 0.002, *P* = 0.313; Φ_SC_ = −0.004, *P* = 0.532, df = 1). The six samples from the western Gulf of Alaska [6–11] were homogeneous (*F*_SC_ = −0.002, *P* = 0.620; Φ_SC_ = −0.00004, *P* = 0.459, df = 5). As with SNPs, the addition of [4, 5] to this group did not produce significant heterogeneity (*F*_SC_ = 0.005, *P* = 0.095; Φ_SC_ = −0.001, *P* = 0.527, df = 7). However, a comparison of haplotype frequencies between samples [4, 5] and [6–11] was marginally significant (*F*_CT_ = 0.016, *P* = 0.041, df = 1), but not for frequencies plus haplotype divergences (Φ_SC_ = 0.005, *P* = 0.432, df = 1). In Southeast Alaska, a core group [12–14, 16, 17] was genetically homogeneous (*F*_SC_ = −0.001, *P* = 0.519; Φ_SC_ = −0.001, *P* = 0.261, df = 4), but the inclusion of sample 15 from Seymour Canal to the group produced a significant amount of heterogeneity (*F*_SC_ = 0.357, *P* < 0.00001; Φ_SC_ = 0.400, *P <* 0.00001, df = 5).

**Figure 5:**
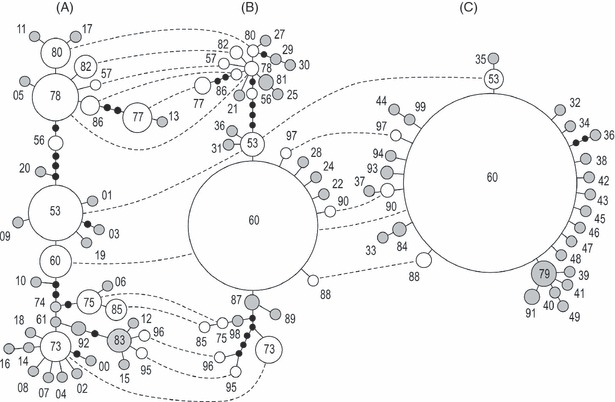
Parsimony haplotype networks for three major evolutionary groups of red king crabs in the North Pacific and Bering Sea. (a) Okhotsk Sea-Norton Sound-Adak Island, (b) southeastern Bering Sea and western Gulf of Alaska, and (c) Southeast Alaska. Open circles represent observed haplotypes, and closed circles represent hypothetical, unobserved haplotypes. Gray circles represent private haplotypes, appearing only in a particular group. Dashed lines trace common haplotypes among groups. Haplotype labels are the last two digits of haplotypes GenBank accession numbers JF738153–JF38249.

The haplotype networks (Fig. 5) varied considerably among regions. The haplotype networks for the western group [1–3] were complex, lacking a central, high-frequency haplotype. The network for combined samples from the southeastern Bering Sea and Gulf of Alaska [6–11] (*n* = 551) had a central haplotype (JF738160) that occurred in 57.2% of the crabs. In the haplotype network for the combined Southeast Alaska samples [12–17], JF738160 occurred in 80.7% of the crabs. Several low-frequency haplotypes, unique to the region, were one substitution from the common haplotype. A unique small lineage appeared in Seymour Canal [15], in which the central haplotype was one mutation removed from the most common haplotype.

The shapes of the mismatch distributions corresponded to the complexities of the haplotype networks for the three groups (Table S7, Figs S1–S3). Western Pacific and northern Bering Sea samples [1, 2, 3] showed broad mismatch distributions with mean mismatches larger than 5.30 (Table S7, Fig. S1), whereas southeastern Bering Sea and western Gulf of Alaska samples showed ragged distributions with mean mismatches between 3.09 and 4.46 (Table S7, Fig. S2). Samples from Southeast Alaska showed strongly unimodal distributions with mean mismatches between 0.12 and 0.36 (Table S7, Fig. S3). Tests of neutrality followed these regional differences. Tajima’s *D*_T_ was not significant in samples from the western Pacific, Bering Sea, and western Gulf of Alaska, but was significant and negative for each of the samples from Southeast Alaska, indicating excesses of low-frequency mutations (Table 1).

**Figure 6:**
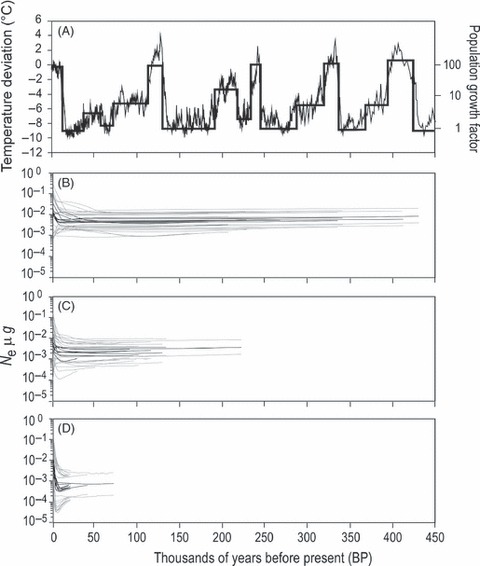
Simulations of historical demography in red king crab. (a) Late Pleistocene demographic model based on climate temperatures estimated from oxygen isotopes in Antarctic ice cores (Jouzel et al. 2007). Effective population sizes vary over two orders of magnitude between glacial maxima and interglacial warmings. (b) Simulations of NW Pacific and N Bering Sea populations. Bayesian skyline plots (BSPs) of 10 replicate samples of sequences (*n* = 200 for each sample) simulated with the program Mesquite, using coalescence, and assuming a base effective population size of *N*_e_ = 800 000 and a scaling factor (approximate mutation rate) = 5 × 10^−9^. Bold curves present the Bayesian estimates of historical population size, and gray curves present the 95% highest probability densities. (c) Simulations of SE Bering Sea and western Gulf of Alaska populations. BSPs of 10 replicate simulated samples as in 6b, except *N*_e_ = 400 000 and scaling factor = 3 × 10^−9^. (d) Simulations of SE Alaska populations. BSPs of 10 replicate simulated samples as in 6b, except *N*_e_ = 100 000.

**Figure 7:**
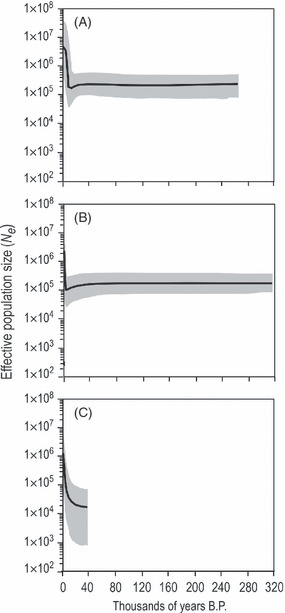
Bayesian skyline plots (BSPs) of three major evolutionary groups of red king crab in the North Pacific and Bering Sea. Substitution rate was estimated to be 5 × 10^−9^ from the position of the postglacial inflection in the population growth curve and was used to scale *N*_e_ on the *y*-axis. (a) Okhotsk Sea–Norton Sound–Adak Island, (b) SE Bering Sea and western Gulf of Alaska, (c) SE Alaska.

The accuracies of inferences about the timings of population events and estimates of population size from the BSPs depend on a reliable molecular-clock calibration. Because no lineage-specific calibration is available for Lithodid crabs, we estimated a mutation rate in two steps. First, we constructed BSPs from the simulated sequences, and these BSPs depicted only the last postglacial population expansion (Fig. 6). The long period of apparent population stability in the simulated BSPs is similar to that in the observed BSPs. These flat demographies represent a loss of historical information before the LGM (Fig. 7). Second, we assumed that the observed episode of population growth in red king crab began about 12 000 years ago, when coastal habitats became available for colonization. This date, together with a generation time of 5 years, yielded a per-year, per-site mutation rate of about 5 × 10^−9^. Historical values of *N*_e_, estimated from *N*_e_μ*g*, indicated that each group of red king crabs experienced a postglacial expansion of at least an order of magnitude (Table 4). For example, the BSP of the Asian-W Bering Sea group depicted an expansion from *N*_e_ = 180 000 to 5 560 000 crabs, and the BSP for the SE Alaska group showed an expansion from *N*_e_ = 84 000 to 2 188 000 crabs.

**Table 4:** Estimates of historical demography from Bayesian skyline plots.*

Major group	Sample size	LGM *N*_e_	Contemporary *N*_e_	95% HPD
NW Pacific–N Bering Sea	213	1.80 × 10^5^	5.56 × 10^6^	6.40 × 10^4^–2.98 × 10^7^
SE Bering–W Gulf of Alaska	551	1.16 × 10^5^	2.20 × 10^6^	1.56 × 10^4^–1.23 × 10^7^
SE Alaska	515	8.40 × 10^4^	2.19 × 10^6^	6.36 × 10^4^–2.98 × 10^6^

* Generation time = 5 years; substitution rate = 5 × 10^−9^ derived from BSPs.

## Discussion

Previous genetic studies of red king crabs in the Alaskan waters did not resolve some details of population structure reported here. The SNP and mtDNA markers and samples sizes used in this study can detect low levels of differentiation among populations (*F*_ST_ = 0.01) with statistical power of 95%, or greater. However, these markers provide only a small amount of power (10–50%) to detect finer differences among populations (*F*_ST_ = 0.001). Such small differences likely reflect short-term frequency shifts from unequal recruitment among populations, or temporary isolations stemming from North Pacific ocean–climate regime shifts. Hence, our discussion is focused on larger-scale geographic patterns resulting from glacial isolations, postglacial dispersals, and population expansions.

The results of this study reveal two important features of red king crab population structure. First, mtDNA diversity varies clinally across the North Pacific, with high levels of diversity in the western North Pacific and progressively lower diversities in eastern populations. Second, tests of SNP and mtDNA frequencies resolved three major evolutionary groups of populations. These features of red king crab population structure appear to reflect contemporary patterns of connectivity influenced by oceanic processes that are superimposed on a Pleistocene history of isolations in refugia and postglacial colonizations. The challenge confronting fishery biologists is how to use these results to improve the management of a declining red king crab resource.

### Diversity gradient across the North Pacific

A striking feature of red king crab phylogeographic structure is a strong gradient in both mtDNA haplotype and nucleotide diversity across the North Pacific. While some SNPs show allele-frequency clines across the North Pacific, a comparable drop in diversity within populations was absent, most likely because SNPs are assays of specific nucleotides and, hence, are not suitable for surveying geographic patterns of within-population diversity. Western populations have high levels of mtDNA diversity (*h* = 0.833–0.911, Θ_π_ = 0.80–0.84%) that are typical of many marine species (Grant and Bowen 1998). High levels of diversity were also found in Russian populations of red king crabs (Zelenina et al. 2008). In contrast, populations in the southeastern Bering Sea and western Gulf of Alaska had lower levels of mtDNA diversity (*h* = 0.556–0.751, Θ_π_ = 0.46–0.67%), and populations in Southeast Alaska had even lower diversities (*h* = 0.118–0.314, Θ_π_ = 0.02–0.05%).

The strong longitudinal gradient in diversity in red king crabs is unusual among large crustaceans. Other crustaceans show gradients in diversity, but these gradients are correlated with latitude (Table S8; Triantafyllidis et al. 2005) or with particular islands (McMillen-Jackson and Bert 2004; Roman and Palumbi 2004). Drops in diversity in island populations, especially islands previously covered with tidewater glaciers, are likely due to founder effects from stepping-stone colonizations (Hewitt 2000). The longitudinal gradient in red king crabs may reflect one of two scenarios. In one, population fragmentation and commercial harvests in the eastern North Pacific over several decades have reduced effective population sizes to an extent that diversity has been lost through genetic drift in some regions. Overharvesting has been invoked to explain low genetic diversities in some marine species (Hauser et al. 2002). The small populations in Southeast Alaska (Clark et al. 2003; SAFE Plan Team 2010) may have been particularly vulnerable to the effects of harvesting, even though harvest rates have not been as large as those in Bristol Bay and the western Gulf of Alaska. Populations in Norton Sound, the southeastern Bering Sea, and western Gulf of Alaska have been heavily harvested, but these populations do not show depressed levels of genetic diversity. Hence, the reduced genetic diversities in Southeast Alaska may reflect, in part, high levels of random drift in small populations inhabiting semi-enclosed fjords.

A second, more likely, scenario invokes the influence of Pleistocene climate variation on red king crab populations. Major glaciations have occurred about every 100 000 years (Milankovitch cycles) for the last 800 000 years (Imbrie et al. 1984). Each cycle of cooling produced coastal glaciers that extirpated populations of near-shore marine species in the Northeast Pacific (Mann and Hamilton 1995; Barrie and Conway 1999). Red king crab populations are especially vulnerable to glaciated coastlines, because shallow-water nursery areas are needed to complete the crab’s life-history cycle (Shirley and Shirley 1989b; Gabaev 2007). The last glaciation reached a maximum about 18 kyr ago, but most of the coastal areas along the Northeast Pacific were not suitable for colonization until 15 kyr ago, or until after the Younger Dryas climate reversal 12–11 kyr ago (Mix et al. 1999).

**Figure 8:**
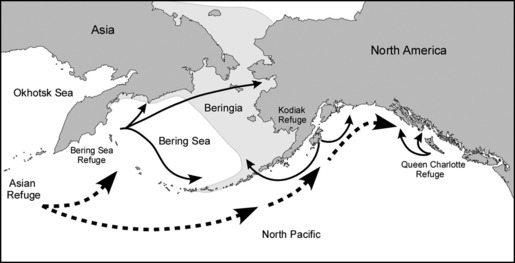
Map of the North Pacific Ocean and Bering Sea showing hypothesized postglacial dispersal routes. Thick dashed lines represent long-term dispersals from an Asian glacial refuge into the Bering Sea and northeastern Pacific. Thin lines represent dispersals from glacial refugia after the last glacial maximum (LGM) 18 kyr ago. Light shading represents the extent of the Bering Land Bridge at the LGM. Colonizations of coastal areas in the northeastern Pacific were not possible until 15 kyr ago or until after the Younger Dryas climate reversal at 12–11 kyr. Colonization of Norton Sound and Bristol Bay was not possible until about 11 kyr after rising sea levels flooded the Bering Land Bridge.

The high levels of genetic diversity, ragged mismatch distributions, and long coalescence times in the northwestern Pacific indicate that these populations have remained large over the Pleistocene and likely supplied colonists for extirpated eastern North Pacific areas on long time scales (dashed line in Fig. 8). However, the prevalence of private alleles in each group indicates that present-day populations were founded by colonists from three local refugia. Western populations likely arose from a glacial refuge population located in a smaller western Bering Sea (Katsuki and Takahashi 2005) or in the Sea of Japan (Ikehara and Itaki 2007). A close tie between populations in the Sea of Japan and around the Kamchatka Peninsula is indicated by a lack of mtDNA and microsatellite differentiation between populations in these regions (Zelenina et al. 2008). A second refuge may have been located around Kodiak Island, which remained unglaciated, in part, during the LGM (Karlstrom and Ball 1969). Populations dispersing from this refuge population show ragged mtDNA mismatch distributions and no departures from neutrality, indicating that the refuge population was large and likely persisted over several glacial cycles. A third refuge may have been located around the Queen Charlotte Islands, which were partially ice free and served as a glacial refuge for terrestrial species (O’Reilly et al. 1993; Bryun et al. 1997). Red king crab populations in Southeast Alaska are genetically imprinted with a signature of a recent expansion indicated by significantly negative values of Tajima’s *D*_T_, by a unimodal mismatch distribution with a mean number of mismatches <1.0, and by a large number of private haplotypes.

The Bayesian skyline plots of both the observed and simulated sequences provide insights into the timing of the population expansions and the limitations of coalescence analysis. First, the timing of recent population growth in each major group is associated with postglacial warming when large expanses of coastal habitats opened to colonization. Second, coalescence analysis of the observed and simulated sequences was unable to resolve population histories prior to the last glacial cycle. Small population sizes during the LGM appear to have erased information about previous histories, even though population sizes were large enough to preserve high levels of genetic diversity in some populations. This loss of information about demographic histories before the LGM has not been previously recognized in the interpretations of BSPs.

Third, the use of an appropriate molecular-clock calibration greatly influences the accuracy of conclusions from coalescence analysis (Ho et al. 2011). Many studies have used mutation rates based on species’ divergences dated by a geological event, such as the closure of the Isthmus of Panama, or the opening of the Bering Strait. These calibrations have led to unlikely scenarios that place population expansions at, or before, the LGM (Carr and Marshall 2008; Ruzzante et al. 2008; Canino et al. 2010; Liu et al. 2011). However, the simulations here show that the pervasive signal of recent population growth in marine organisms likely reflects postglacial expansions. These results underscore the need for internally, or empirically, calibrated substitution rates (Ho et al. 2008; Crandall et al. 2012). Nevertheless, the population-level molecular-clock rate estimated here is slow compared to time-dependent calibrations for other species (Ho et al. 2008). This low rate may indicate that red king crabs have an intrinsically low rate of genomic mutation and may explain the low levels of variability for some classes of molecular markers (Grant et al. 2011).

### Contemporary genetic population structure

On short time scales, the effects of larval dispersals in ocean currents play out against an ice-age legacy. High levels of gene flow by larval drift are expected to produce geographically large populations (Waples 1998), while isolation by shoreline complexity and mesoscale eddies is expected to produce a more subdivided population structure (Gunderson and Vetter 2006). In the present study, SNP and mtDNA variability define at least three regional population groups that vary in size. However, the processes influencing population structure within the groups appear to differ, because of differences among regions in oceanic and shoreline barriers to dispersal.

Managers are challenged to distinguish equilibrium from nonequilibrium influences on geographic patterns of genetic variability. Recent expansions from ice-age refugia may produce the appearance of genetic homogeneity among populations, which, in fact, may be demographically isolated from one another. Genetic homogeneity may persist, because divergence from random drift is minimal in large populations. This mechanism has been invoked to explain genetic homogeneity among populations of American lobsters (Kenchington et al. 2009), European lobsters (Triantafyllidis et al. 2005), Norwegian lobsters (Stamatis et al. 2004), and spiny lobsters (Tolley et al. 2005). Like red king crabs, these species have star-shaped mtDNA haplotype genealogies indicative of a recent population expansion. Despite the potential homogenizing effects of postglacial expansions, genetic similarity among populations has been explained solely by high levels of gene flow in spiny spider crabs (Sotelo et al. 2008) and green crab (Domingues et al. 2010). Gene flow in pelagic crustacean such as Antarctic krill (Bortolotto et al. 2011) is undoubtedly responsible for genetic homogeneity among populations.

#### Southeast Alaska

Even though these populations represent a single evolutionary unit, some populations appear to be isolated from other populations, indicating demographic independence. In an extreme case, Seymour Canal is nearly fixed for a unique mtDNA lineage, indicating virtually no connection with other Southeast Alaska populations (Fig. 3b, Table 3). These isolations may reflect barriers to dispersal imposed by shoreline configurations and mesoscale eddies (Hermann et al. 2002; Weingartner et al. 2009). Southeast Alaska populations also show a fivefold drop in haplotype diversity and over an order of magnitude drop in nucleotide diversity from diversities in other regions. These low levels of genetic diversity may impede adaptive responses to environmental changes and may predispose these populations to extinctions from climate shifts. The genetic results support the present management practice of using a regional guideline to set harvest limits, and closing small areas to harvests when local populations are reduced. Any stock enhancements in Southeast Alaska must carefully consider the geography of genetic variability among populations and match the genetics of released crabs with the genetic characteristics of the local populations.

#### Bering Sea

Red king crab populations at the Arctic edge of the distribution in the Bering Sea are also genetically heterogeneous, indicating demographic independence from one another. The western Aleutian and Norton Sound crabs appear to be isolated from southeastern Bering Sea populations, despite ocean currents that would be expected to promote gene flow between populations (Fig. 2a). Norton Sound red king crabs differ from crabs in southern areas in such life-history traits as size and age at maturity, and recruitment (Zheng and Kruse 2000; SAFE Plan Team 2010). The biology of early life-history stages and juveniles that takes place under ice is largely unknown. Life-history differences may reflect a legacy of Pleistocene isolations in a smaller western Bering Sea that was ecologically similar to Norton Sound (Brigham-Grette 2001). Adaptive responses to contemporary environmental selection may reinforce isolation between Norton Sound and southeastern Bering Sea populations, because hybrids derived from migrants and local crabs may not survive in northern waters.

Bristol Bay (including the Pribilof Islands) red king crabs, on the other hand, are at the extremes of a large group of genetically homogeneous populations in the northern Gulf of Alaska. Southeastern Bering Sea populations are tied to northern Gulf of Alaska populations by recent common ancestry and by dispersals from the western Gulf of Alaska. Other species, including flatfishes (Best 1977; St-Pierre 1989; Bailey and Picquelle 2002) and Walleye pollock (Bailey et al. 1999), show linkages between the western Gulf of Alaska and southeastern Bering Sea. Despite the genetic and life-history similarities between Bristol Bay and Gulf of Alaska populations, these two groups are demographically independent of one another. While the Bristol Bay red king crab population recovered from population crashes in the 1970s and 1980s and supports a fishery today, the Kodiak Island fishery in the Gulf of Alaska remains closed (Bechtol and Kruse 2009). Hence, occasional larval dispersals from the Gulf of Alaska may limit genetic divergence, but does not exceed the critical number of migrants needed to produce a common demography with Bristol Bay populations (Waples and Gaggiotti 2006).

#### Western Gulf of Alaska

The genetically homogeneous populations in this region may have spread from the Kodiak refuge. Parts of the haplotype network for this group consist of a star-shaped mtDNA genealogy, indicating a recent population expansion. It is uncertain, however, whether the genetic similarity among populations is a legacy of the expansion or whether these populations are connected by high levels of larval dispersal in the fast-moving Alaska Coastal Current (Hermann et al. 2002; Fig. 2a). The Alaska Coastal Current narrows and feeds the fast-moving southwestward Alaska Stream, which flows along the southern edge of the Alaska Peninsula toward the Aleutian Island chain (Stabeno et al. 2001). Larvae may be carried into the Southeastern Bering Sea and into Bristol Bay, but the genetic dissimilarity between Gulf of Alaska and Aleutian Island populations indicates that larvae are not carried into the Aleutian Island chain. This is consistent with a boundary at Samalga Pass marking a biogeographical transition (Hunt and Stabeno 2005).

Other information from stock assessments may provide insights into genetic population structure. Synchronous responses among red king crab populations to climate variables may reflect genomic similarities. For example, populations in the southeastern Bering Sea and western Gulf of Alaska showed similar recruitment patterns over several decades, but these patterns were significantly different from those in Norton Sound and Southeast Alaska (Zheng and Kruse 2000). In recent years, differences in abundances between Bristol Bay and Kodiak Island populations are clear indications of a lack of ecological connectivity between populations in those areas.

### Implications for management

The results of this study clarify issues in the management of red king crab harvests and possible stock enhancements. The genetically defined groups of red king crabs largely coincide with the State of Alaska’s registration areas. Populations in the northeastern Bering Sea (Q), Bristol Bay (T), and the Aleutian Islands (O) are genetically divergent from one another, because of different biogeographical histories. However, oceanic current patterns or biotic interactions prevent large-scale mixing between these populations. The genetic discontinuity between western Aleutian and southeastern Bering Sea populations coincides with a biogeographical boundary at Samalga Pass in the Aleutians that has been attributed to oceanic features (Hunt and Stabeno 2005). Additionally, biotic interactions between the divergent populations may also prevent mixing between populations. High density blocking (incumbency), or competitive exclusion (Waters 2011), may prevent secondary dispersers from becoming established in areas already occupied by red king crabs.

A genetically homogeneous group of red king crabs includes populations in the southeastern Bering Sea (T) and western Gulf of Alaska (M, K, H), and possibly the northern Gulf of Alaska (E, D). Genetic similarity among these populations likely reflects a postglacial radiation from a common glacial refuge and may not indicate close ties by gene flow. While no overall significance was detected among populations in this group, significant SNP and mtDNA divergence was detected with *F*_ST_, which is based on frequencies, but not with Φ_ST_, which additionally incorporates sequence divergences between haplotypes (Tables 2 and 3). Larger haplotype diversities in southeastern Bering populations than in western Gulf of Alaska populations also indicate demographic independence (Table 1). These genetic contrasts, together with different abundance trajectories in the last few decades (Otto 1986; Bechtol and Kruse 2009), indicate that southeastern Bering Sea populations warrant continued management as a separate unit.

In contrast, Southeast Alaskan populations show much greater levels of subdivision. These populations may have expanded into Southeast Alaskan waters from a glacial refuge population, but are presently isolated in semi-enclosed fjords that limit larval dispersal. Private mtDNA haplotypes in all of these populations indicate isolation and population self-recruitment. These populations may also have diverged from one another because of adaptive shifts to local environmental conditions. While all of these populations fall into a single registration area, they are best managed on a finer geographic scale, because of their demographic independence.

Research is underway to develop hatchery techniques to raise crabs for restoring depressed populations, or for stock enhancement. If stock restoration is warranted because of reproductive limitations, several genetic factors must be considered in devising a stock restoration plan. A close genetic match between hatchery broodstock and the depressed population is desirable. While the results of population genetic studies provide important background information, the molecular markers used in these studies are generally neutral to selection and may not detect important adaptive differences among populations. Patterns of gene flow between local populations and the spatial scale of the adaptive landscape for red king crab are unknown. In addition to broodstock choice, artificial culture may alter the genetic profiles of hatchery-reared crabs, because more larvae survive in a hatchery setting than in the wild, setting the stage for selective or random shifts in genetic profiles. Hybridizations between hatchery-reared crabs and wild crabs may compromise the genetic integrity of the wild populations, and persistent hatchery releases may displace wild populations with crabs of hatchery ancestry (Ryman and Laikre 1991).

In conclusion, the distributions of SNP and mtDNA population markers portray the effects of ancient isolations and dispersals, contemporary gene flow, and random drift on population structure. These findings provide managers with a sound basis for managing harvests and restorations on a population level. However, the failure to find significant differences among populations in some areas with neutral molecular markers does not necessarily indicate that populations are connected with large amounts of gene flow. Historical expansions from a common ice-age refuge can produce the appearance of panmixia, even though populations may be demographically independent from one another.

## Data archiving

Data for this study are available at: mitochondrial DNA sequences, GenBank accession numbers JF738153–JF738249.

## Supporting information

**Figure S1.** MtDNA mismatch distributions in samples from western Pacific populations.

**Figure S2.** MtDNA mismatch distributions in samples from the SE Bering Sea and western Gulf of Alaska.

**Figure S3.** MtDNA mismatch distribution in samples from SE Alaska.

**Table S1.** Fifteen single nucleotide polymorphic (SNP) primers and markers in red king crab.

**Table S2.** Allele frequencies for 15 single nucleotide polymorphisms in 17 samples of red king crab from the North Pacific.

**Table S3.** Values of *F*_ST_,based on frequencies of 15 SNP loci, between samples of NorthPacific red king crab.

**Table S4.** Haplotype frequencies for a 665-bp fragment of cytochrome oxidase subunit 1 in red king crab samples from the North Pacific.

**Table S5.** Values of *F*_ST_,based on mitochondrial DNA haplotype frequencies, between NorthPacific red king crab.

**Table S6.** Values of Φ_ST_, basedon mitochondrial DNA frequencies and haplotype divergences, betweensamples of North Pacific red king crab.

**Table S7.** Summary statistics from mismatch distributions.

**Table S8.** Comparison of genetic population structure among crustaceans.
