# Supplementary material for: Incorporating deep and shallow components of genetic structure into the management of Alaskan red king crab
Source: Evol Appl. 2012 Dec;5(8):820–37. doi: 10.1111/j.1752-4571.2012.00260.x (PMC3552400; doi:10.1111/j.1752-4571.2012.00260.x)

Supporting tables and figures for Grant and Cheng, Incorporating historical and contemporary components of genetic structure into the management of Alaskan red king crab. *Evolutionary Applications*

Table S1 Fifteen single nucleotide polymorphic (SNP) primers and markers in red king crab

Table S2 Allele frequencies for 15 single nucleotide polymorphisms in 17 samples of red king crab from the North Pacific

Table S3 Values of F_{ST}, based on frequencies of 15 SNP loci, between samples of North Pacific red king crab

Table S4 Haplotype frequencies for a 665 bp fragment of cytochrome oxidase subunit 1 in red king crabs from the North Pacific

Table S5 Values of F_{ST}, based on mitochondrial DNA haplotype frequencies, between North Pacific red king crab

Table S6 Values of Φ_{ST}, based on mitochondrial DNA frequencies and divergences (Tamura & Nei 1993), between samples of North Pacific red king crab

Table S7 Summary statistics from mismatch distributions

Table S8 Comparison of genetic population structure among crustaceans

Figure S1 MtDNA mismatch distributions in samples from western populations.

Figure S2 MtDNA mismatch distributions in samples from the SE Bering Sea and western Gulf of Alaska.

Figure S3 MtDNA mismatch distribution in samples from SE Alaska.

Table S1. Fifteen single nucleotide polymorphic (SNP) (An *et al.* 2010) primers and markers in red king crab, polymerase chain reaction (PCR) sequences for forward (F) and reverse (R) primers, and 'VIC' and 'FAM' sequences are optical markers of the SNP. Marker names consist of the species identifier (*Pca* for *Paralithodes camtschaticus*) and arbitrary numbers. T_a are optimized annealing temperatures.

Marker name /GenBank accession Number	Oligonucleotide sequences (5'-3')[a]	T_a (^oC)
Pca_U0001<br>GU128158	F: CAACAACAGATGGCGTTCATAATGA<br>R: TCCTATGTAGTAATAATATTGGGACCATAAAAGGT<br>VIC- CCAAGGGATCTCATTTT<br>FAM- AAGGGATCTAATTTT	59
Pca_U0002<br>GU128159	F: TGTCTAAAACAATGCAACTTCAAGCAAA<br>R: TGGCAAGCTAAGACAATGTTGGTTA<br>VIC- TCCCACTCAAACTCAA<br>FAM- ATTCCCACTCATACTCAAA	60
Pca_U0003<br>GU128160	F: ACAATGTTATGCAGTTTAAGAAAGACCTATGT<br>R: CGTATTCTGGTTACCATCTGACGAT<br>VIC- CAAATGAGTATATACAATCT<br>FAM- CAAATGAGTAAATACAATCT	59
Pca_U0004<br>GU128161	F: GGCTGTGAATGTCCACTCCTATT<br>R: GCCCTTTTTGAGATACAGTACAAAGC<br>VIC- CCATTAGGGAAGAGTCA<br>FAM- CATTAGGGAGGAGTCA	58
Pca_U0005<br>GU128162	F: CAGGGATCGACTTCCGCTTAA<br>R: AGCTCTCTGTAGCGAGTAATCGATA<br>VIC- TCAACGGCGCCAGTT<br>FAM- TCAACGGCACCAGTT	58
Pca_U0006<br>GU128163	F: TGCTTGCCTTCCAGTATGTTCAT<br>R: CTCCTGTAGGTCATGCCATCATTAT<br>VIC- CCTGCCAAATTTCTTA<br>FAM- CTGCCAAACTTCTTA	60
Pca_U0007<br>GU128164	F: CATCTCTTTTTCCATTGTATACATTGCACAT<br>R: CCGCAGAGTCTCAGAATATTGTTTAGG<br>VIC- ACATTAACTTTTAAGCTTTTCA<br>FAM- CATTAACTTTTAAGGTTTTCA	60
Pca_U0008	F:	60

	CCAACATACTTAAAATTGTAAACGATAAGAAAAGAACT	
GU128165	R: AGCTTCGGCATGGACGTTAG	
	VIC- ACACTATGATAGAAATAAAA	
	FAM- ACTATGATAGAAAAAAAA	
Pca_U0009	F: GCTCTACATCATAGTAAGGCCAAGTT	60
GU128166	R: TTCAACTGAAGGTAAATTGCAATGTTATCATAAAA	
	VIC- CATATTTCACTACAAAAAC	
	FAM- ATTTCACTCCAAAAAC	
Pca_U0010	F: GCATGTGCGTTTGAGTTTTGTATCT	60
GU128167	R: CCCGGCACCACTTAGGAT	
	VIC- ATGCAGTTGGTAAAGAA	
	FAM- ATGCAGTTGTTAAAGAA	
Pca_U0011	F: ACAGAGCAAGCCAAATGTAATGACA	60
GU128168	R: TGTTTGACGGTACTGGAACGG	
	VIC- TCGCGCTAATAAGTCATGT	
	FAM- TCGCGCTAATAATTCATGT	
Pca_U0012	F: GCGTGATAGTGGGAGGGAAATC	
GU128169	R: ACAAATATTTAAACCTGTTCTTGTCTGCTAGT	60
	VIC- AACAGCTGATAAAAAC	
	FAM- ACAGCTGTTAAAAAC	
Pca_U0013	F: CTTCTCCCGAAACAAGCTAGCTAA	60
GU128170	R: GGTGGCCATGAATGAAACTCCTT	
	VIC- CATCAGGGTAATTAATTTGA	
	FAM- ATCAGGGTAATTCATTTGA	
Pca_U0014	F: ATCAGGGTAATTCATTTGA	60
GU128171	R: CTTATTGGCCAGAGTGGACAGA	
	VIC- CTTATTGGCCAGAGTGGACAGA	
	FAM- CTTATTGGCCAGAGTGGACAGA	
Pca_U0015	F: ACTGACATGCCTATCGATCTATTGC	60
GU128172	R: GGCTACTGTACATGGAGATGATTCA	
	VIC- CATGAGGACCAAGACGT	
	FAM- CATGAGGACCCAGACGT	

[a]Each allele-specific probe was labelled with either VIC or FAM on its 5' end and bore a minor groove binder and a nonfluorescent quencher on its 3' end

Reference

An, J.-H., Bechet, A., Berggren, A., Brown, S.-K. *et al*. 2010. Permanent genetic resources added to Molecular Ecology Resources database 1 October 2009-30 November 2009. *Molecular Ecology Resources* **10**:404–408.

Table S2. Allele frequencies for 15 single nucleotide polymorphisms in 17 samples of red king crab from the North Pacific

	U0001			U0002			U0003			U0004			U0005		
Location	G	T	2*N*	A	T	2*N*	A	T	2*N*	C	T	2*N*	C	T	2*N*
1. Okhostk Sea	0.962	0.038	104	0.000	1.000	104	0.638	0.362	94	0.048	0.952	104	0.888	0.112	98
2. Norton Sound	0.899	0.101	188	0.038	0.962	182	0.586	0.414	186	0.059	0.941	186	0.870	0.130	184
3. Adak Island	0.768	0.232	142	0.019	0.981	162	0.689	0.311	164	0.024	0.976	164	0.873	0 .127	158
4. Probilof Island	0.892	0.108	186	0.016	0.984	186	0.608	0.392	186	0.032	0.968	186	0.853	0 .147	184
5. Bristol Bay	0.878	0.122	180	0.006	0.994	158	0.638	0.362	130	0.006	0.994	168	0.820	0.180	122
6. Alitak Bay	0.962	0.038	184	0.000	1.000	188	0.506	0.494	178	0.016	0.984	184	0.776	0.224	174
7. Chiniak Bay	0.958	0.042	190	0.000	1.000	190	0.574	0.426	188	0.021	0.979	190	0.811	0.189	190
8. Uganik Bay	0.921	0.079	190	0.000	1.000	144	0.560	0.440	168	0.011	0.989	190	0.772	0.228	184
9. Kukak Bay	0.935	0.065	124	0.000	1.000	124	0.526	0.474	114	0.016	0.984	124	0.730	0.270	122
10. Kamishak Bay	0.924	0.076	132	0.000	1.000	128	0.524	0.476	124	0.023	0.977	132	0.800	0.200	130
11. Kachemak Bay	0.940	0.060	84	0.000	1.000	90	0.511	0.489	88	0.033	0.967	90	0.778	0.222	90
12. St James Bay	1.000	0.000	184	0.000	1.000	144	0.392	0.608	176	0.000	1.000	188	0.761	0.239	180
13. Eagle River	1.000	0.000	182	0.000	1.000	188	0.297	0.703	182	0.000	1.000	186	0.694	0.306	186
14. Barlow Cove	1.000	0.000	188	0.000	1.000	184	0.424	0.576	170	0.016	0.984	182	0.741	0.259	166
15. Seymour Canal	1.000	0.000	100	0.000	1.000	98	0.478	0.522	90	0.010	0.990	100	0.773	0.227	88
16. Deadman Reach	0.997	0.003	360	0.000	1.000	382	0.394	0.606	376	0.000	1.000	382	0.761	0.239	380
16a. 1989	1.000	0.000	168	0.000	1.000	190	0.404	0.596	188	0.000	1.000	190	0.753	0.247	190
16b. 2001	0.995	0.005	190	0.000	1.000	192	0.383	0.617	188	0.000	1.000	192	0.768	0.232	190
17. Gambier Bay	1.000	0.000	190	0.000	1.000	190	0.414	0.586	186	0.021	0.979	188	0.739	0.261	188

Table S2. Continued

	U0006			U0007			U0008			U0009			U0010		
	A	G	2*N*	C	G	2*N*	A	T	2*N*	A	C	2*N*	A	C	2*N*
1.	0.010	0.990	102	0.990	0.010	104	0.245	0.755	98	0.794	0.206	102	0.231	0.769	104
2.	0.032	0.968	154	0.984	0.016	182	0.385	0.615	156	0.770	0.230	178	0.247	0.753	182
3.	0.142	0.858	162	1.000	0.000	166	0.111	0.889	162	0.753	0.247	162	0.256	0.744	160
4.	0.092	0.908	184	0.984	0.016	184	0.207	0.793	184	0.837	0.163	184	0.185	0.815	130
5.	0.065	0.935	186	0.982	0.018	164	0.106	0.894	94	0.761	0.239	134	0.145	0.855	138
6.	0.043	0.957	188	0.956	0.044	182	0.187	0.813	182	0.871	0.125	184	0.118	0.882	136
7.	0.068	0.932	190	0.978	0.022	186	0.204	0.796	186	0.830	0.170	182	0.134	0.866	142
8.	0.059	0.941	186	0.984	0.016	186	0.181	0.819	182	0.933	0.067	180	0.170	0.830	188
9.	0.073	0.892	130	0.968	0.032	124	0.196	0.804	112	0.831	0.169	118	0.217	0.783	120
10.	0.108	0.892	130	0.977	0.023	132	0.189	0.811	122	0.850	0.150	80	0.175	0.825	120
11.	0.078	0.922	90	1.000	0.000	90	0.167	0.833	90	0.798	0.202	84	0.179	0.821	56
12.	0.011	0.989	186	0.933	0.067	180	0.262	0.738	172	0.854	0.146	164	0.076	0.924	184
13.	0.005	0.995	186	0.947	0.053	188	0.256	0.744	180	0.844	0.156	180	0.078	0.922	180
14.	0.011	0.989	190	0.962	0.038	184	0.254	0.746	114	0.783	0.217	166	0.086	0.914	174
15.	0.000	1.000	100	0.948	0.052	96	0.152	0.848	66	0.857	0.143	84	0.065	0.935	92
16.	0.008	0.992	382	0.953	0.047	382	0.237	0.763	372	0.841	0.159	328	0.106	0.894	368
16a.	0.011	0.989	190	0.942	0.058	190	0.245	0.771	184	0.863	0.137	168	0.120	0.880	184
16b.	0.005	0.995	192	0.964	0.036	192	0.229	0.806	188	0.819	0.181	160	0.092	0.908	184
17.	0.005	0.995	190	0.932	0.068	190	0.194	0.806	180	0.889	0.111	180	0.079	0.921	190

Table S2. Continued.

	U0011			U0012			U0013			U0014			U0015		
	A	C	2*N*	A	T	2*N*	G	T	2*N*	C	T	2*N*	G	T	2*N*
1.	0.154	0.846	104	0.990	0.010	104	0.596	0.404	104	0.150	0.850	100	0.048	0.952	104
2.	0.200	0.800	170	0.979	0.021	188	0.521	0.479	146	0.176	0.824	188	0.000	1.000	184
3.	0.218	0.782	156	1.000	0.000	164	0.443	0.557	158	0.120	0.880	166	0.037	0.963	164
4.	0.176	0.824	188	0.984	0.016	186	0.415	0.585	188	0.239	0.761	188	0.032	0.968	188
5.	0.178	0.822	180	0.988	0.012	166	0.456	0.544	180	0.185	0.815	178	0.056	0.944	180
6.	0.133	0.867	188	0.995	0.005	184	0.489	0.511	188	0.256	0.744	176	0.070	0.930	186
7.	0.116	0.884	190	0.989	0.011	190	0.405	0.595	190	0.237	0.763	186	0.053	0.947	190
8.	0.132	0.868	190	0.995	0.005	186	0.394	0.606	188	0.253	0.747	190	0.048	0.952	186
9.	0.185	0.815	124	0.992	0.008	124	0.426	0.574	122	0.266	0.734	124	0.024	0.976	124
10.	0.146	0.854	130	1.000	0.000	132	0.518	0.482	114	0.230	0.770	122	0.077	0.923	130
11.	0.179	0.821	84	1.000	0.000	90	0.432	0.568	88	0.256	0.744	86	0.045	0.955	88
12.	0.290	0.710	186	0.995	0.005	186	0.290	0.710	186	0.134	0.866	186	0.215	0.785	186
13.	0.328	0.672	180	1.000	0.000	190	0.303	0.697	188	0.065	0.935	184	0.207	0.793	188
14.	0.228	0.772	184	1.000	0.000	182	0.271	0.729	188	0.119	0.881	176	0.218	0.782	188
15.	0.388	0.612	98	1.000	0.000	98	0.380	0.620	100	0.112	0.888	98	0.280	0.720	100
16.	0.316	0.684	348	1.000	0.000	378	0.305	0.695	308	0.062	0.938	372	0.294	0.706	364
16a.	0.303	0.667	188	1.000	0.000	190	0.337	0.663	184	0.064	0.936	188	0.299	0.701	184
16b.	0.331	0.669	160	1.000	0.000	188	0.258	0.742	124	0.060	0.940	184	0.289	0.711	180
17.	0.266	0.734	188	1.000	0.000	188	0.279	0.721	190	0.087	0.913	184	0.298	0.702	188

Table S4. Haplotype frequencies for a 665 bp fragment of cytochrome oxidase subunit 1 in red king crab samples from the North Pacific and marginal seas. Haplotype designations are the last two digits of the GenBank Accession numbers JF738153–JF38249.

	Location																	
Hap	1	2	3	4	5	6	7	8	9	10	11	12	13	14	15	16a	16b	17
60	1	6	6	46	23	57	53	59	38	14	25	83	62	57		75	79	59
53	10	5	27	12	11	9	5	8	8	2	3	5	1	3		4	3	2
73	9	3	4	13	3	10	13	5	4	5	5							
77	9	11	17	5	3	4	1	6	2		3							
78	4	3	9	6	1	3	3	8	3	1	5							
79															33			
80	1	17	6		1	1			2									
81				1	3	4	4	3	2	3	1							
82	1	10		3		3	1		1	1								
75	1	1	5		1													
83		8	1															
84												2	1			3	1	1
85	1	3	2		1													
56	1	1									1							
86	2	3		1														
87				3		1	1				1							
88																3	3	
89					1			2		1								
90									1					1			1	1
91															4			
57		1		1														
92		3																
93												1				1	1	
94																1	2	
95			1	1														
96			1	1														
97				1								1						
98				1				1										
99													1					1
00	1																	
01	1																	
02	1																	

Location																	
Hap	1	2	3	4	5	6	7	8	9	10	11	12	13	14	15	16a	16b	17
03	1																	
04	1																	
05	1																	
06	1																	
07	1																	
08	1																	
61	1																	
74		1																
09		1																
10		1																
11		1																
12		1																
13		1																
14		1																
15		1																
16		1																
17			1															
18			1															
19			1															
20			1															
21					1													
22					1													
23					1													
24						1												
25						1												
26							1											
27								1										
28								1										
29										1								
30										1								
31											1							
32												1						
33												1						
34													1					
35														1				
36														1				
37														1				

Location																		
Hap	1	2	3	4	5	6	7	8	9	10	11	12	13	14	15	16a	16b	17
38														1				
39															1			
40															1			
41															1			
42																1		
43																1		
44																1		
45																		1
46																		1
47																		1
48																		1
49																		1

Table S3 Values of F_{ST}, based on frequencies of 15 SNP loci, between samples of North Pacific red king crab. Sample numbers as in Table 1.

2	0.0030															
3	0.0232	0.0283														
4	0.0110	0.0113	0.0123													
5	0.0112	0.0183	0.0063	0.0009												
6	0.0167	0.0251	0.0409	0.0067	0.0106											
7	0.0154	0.0192	0.0277	-0.0009	0.0043	0.0004										
8	0.0253	0.0283	0.0328	0.0020	0.0106	0.0011	-0.0003									
9	0.0180	0.0166	0.0261	0.0010	0.0065	0.0004	0.0003	-0.0007								
10	0.0073	0.0149	0.0226	0.0007	0.0036	-0.0032	-0.0001	0.0011	-0.0018							
11	0.0149	0.0168	0.0216	-0.0016	0.0013	-0.0024	-0.0032	-0.0004	-0.0074	-0.0051						
12	0.0677	0.0584	0.0788	0.0434	0.0495	0.0336	0.0355	0.0373	0.0337	0.0390	0.0299					
13	0.0912	0.0795	0.1019	0.0691	0.0719	0.0529	0.0601	0.0598	0.0510	0.0587	0.0486	0.0008				
14	0.0636	0.0548	0.0718	0.0407	0.0427	0.0333	0.0302	0.0364	0.0312	0.0369	0.0257	-0.0023	0.0057			
15	0.0639	0.0650	0.0694	0.0483	0.0456	0.0406	0.0461	0.0478	0.0422	0.0423	0.0360	0.0044	0.0123	0.0103		
16	0.0756	0.0729	0.0856	0.0597	0.0625	0.0494	0.0521	0.0543	0.0506	0.0521	0.0450	0.0004	0.0031	0.0032	0.0021	
17	0.0807	0.0785	0.0866	0.0562	0.0598	0.0441	0.0467	0.0462	0.0473	0.0493	0.0427	-0.0001	0.0058	0.0019	0.0031	-0.0009
	1	2	3	4	5	6	7	8	9	10	11	12	13	14	15	16

Tests of each value for significance from 0.0 are routinely made, but this procedure violates the simple requirement of independence when multiple tests are made of the same samples (e.g. 1x2, 1x3, 1x4 . . . 1xn). Often too, the probabilities of the tests are modified to account for the increase in type I error with repeated tests of the same hypothesis. However, these procedures may not adequately control for false positives or may produce false negatives for some comparisons. Finally, and most importantly, the hypotheses being tested changes across the table. The hypothesis for samples from adjacent locations might be whether the samples were drawn from the same randomly mating population. This is a biologically meaningful hypothesis. However it is better tested with hierarchical AMOVAs in a framework that encourages orthogonal comparisons. Hypotheses of tests between samples drawn from geographically distant populations are more difficult to frame. These hypotheses must also include migration or historical isolation to be biologically realistic. While tests of pairwise comparisons may show statistical significance, it is not always possible to assign biological significance to the results of these tests. Hence, tests of significance for the pairwise divergences are not included in the following three tables.

Table S5. Values of F_{ST}, based on mitochondrial DNA haplotype frequencies, between North Pacific red king crab. Sample numbers as in Table 1.

2	0.0332															
3	0.0057	0.0576														
4	0.1144	0.1278	0.1325													
5	0.0936	0.1119	0.0958	0.0018												
6	0.1930	0.1866	0.2052	0.0046	0.0187											
7	0.2287	0.2192	0.2447	0.0164	0.0438	-0.0051										
8	0.2084	0.2012	0.2140	0.0147	0.0275	-0.0031	0.0048									
9	0.1946	0.1854	0.1983	0.0077	0.0134	-0.0095	0.0002	-0.0077								
10	0.1175	0.1227	0.1499	0.0075	0.0045	0.0007	0.0052	0.0182	0.0107							
11	0.1608	0.1633	0.1779	0.0030	0.0179	-0.0076	-0.0029	-0.0098	-0.0067	-0.0023						
12	0.4673	0.4044	0.4373	0.1616	0.2098	0.0956	0.0938	0.0844	0.0931	0.2206	0.1394					
13	0.5008	0.4257	0.4658	0.1977	0.2618	0.1325	0.1303	0.1207	0.1410	0.2845	0.1910	0.0028				
14	0.4285	0.3712	0.4059	0.1433	0.1851	0.0827	0.0809	0.0725	0.0792	0.1924	0.1198	0.7478	0.0040			
15	0.3781	0.3477	0.3883	0.4386	0.4538	0.5045	0.5409	0.5178	0.5291	0.4898	0.5196	-0.0057	0.8031	0.7343		
16	0.4847	0.4274	0.4600	0.1658	0.2129	0.0928	0.0889	0.0807	0.0882	0.2097	0.1310	0.0028	0.0110	-0.0034	0.7210	
17	0.4124	0.3575	0.3947	0.1323	0.1721	0.0730	0.0703	0.0630	0.0694	0.1711	0.1043	0.7478	0.0075	-0.0087	0.7116	-0.0042
	1	2	3	4	5	6	7	8	9	10	11	12	13	14	15	16

Table S6. Values of Φ_{ST}, based on mitochondrial DNA frequencies and divergences (Tamura & Nei 1993), between samples of North Pacific red king crab. Sample numbers as in Table 1

2	0.0341															
3	0.0012	0.0402														
4	0.1040	0.2047	0.0839													
5	0.1253	0.2222	0.0933	-0.0045												
6	0.1568	0.2543	0.1236	-0.0016	-0.0089											
7	0.1869	0.2903	0.1593	0.0047	0.0053	-0.0026										
8	0.1583	0.2475	0.1156	0.0038	-0.0030	-0.0044	0.0095									
9	0.1674	0.2579	0.1266	0.0014	-0.0094	-0.0110	-0.0006	-0.0108								
10	0.0549	0.1457	0.0486	-0.0109	-0.0024	0.0036	0.0186	0.0152	0.0148							
11	0.1000	0.1912	0.0716	-0.0093	-0.0047	-0.0037	0.0109	-0.0105	-0.0062	-0.0052						
12	0.5291	0.5434	0.4202	0.1985	0.2430	0.1633	0.1505	0.1610	0.1782	0.3872	0.2664					
13	0.4924	0.5115	0.3924	0.1862	0.2290	0.1543	0.1426	0.1513	0.1683	0.3591	0.2448	-0.0003				
14	0.4633	0.4927	0.3696	0.1655	0.1926	0.1322	0.1207	0.1302	0.1379	0.3141	0.2115	0.0034	0.0100			
15	0.4228	0.4335	0.3467	0.2683	0.3243	0.2745	0.3103	0.2577	0.3056	0.3657	0.3053	0.7848	0.8334	0.7398		
16	0.6265	0.6290	0.5091	0.2604	0.3192	0.2162	0.2019	0.2143	0.2394	0.4822	0.3510	-0.0002	-0.0001	0.0072	0.7691	
17	0.4837	0.5075	0.3869	0.1795	0.2155	0.1468	0.1338	0.1448	0.1574	0.3405	0.2327	-0.0020	-0.0070	-0.0002	0.7664	0.0002
	1	2	3	4	5	6	7	8	9	10	11	12	13	14	15	16

Reference

Tamura, K., and M. Nei. 1993. Estimation of the number of nucleotide substitutions in the control region of mitochondrial DNA in humans and chimpanzees. *Molecular Biology and Evolution* **10**: 512–526.

Table S7. Comparison of genetic population structure among crustaceans

Species	km	S	n	h	Mitochondrial DNA Θ_π	Mitochondrial DNA F_{ST}	Mitochondrial DNA F_{SC}	Microsatellites No.	Microsatellites F_{ST}	Microsatellites F_{SC}	Comment	Ref.[d]
Red king crab (*Paralithodes camtschaticus*)	4100	17	1278	0.911<br>0.662<br>0.236	0.0085<br>0.0031<br>0.0004	0.139	0.124	15[e]	0.054	0.005	Strong diversity gradient across North Pacific. Large amount of divergence among regional groups.	1
Spiny spider crab (*Maja brachydactyla*)	3000	15	299	0.819	0.0038	0.038	–	9	0.009	–	Occurs in shallow water (0–100m), No large-scale differentiation, but local isolation	2
Green crab	4200	18	1482	–	–	–	–	10	0.001[c]	0.0001	3 groups, no isolation by distance (IBD)	3
(*Carcinus maenas*)	3300	14	217	0.855<br>0.280	0.0047<br>0.0016	0.170	0.030	–	–	–	Strong divergence between island and continental populations. Little divergence among continental populations. Drop in island diversity. No IBD.	4
American lobster (*Homarus americanus*)	1860	35	2555	–	–	–	–	13	0.006[c]	–	North-south groups. Ice-age isolation and bifurcating currents.	5
European lobster (*Homarus gammarus*)	7100	44	3283	0.517<br>0.912<br>0.610<br>0.907<br>0.846<br>0.551	–	0.111	0.007	–	–	–	5 groups. No differentiation among 24 central Atlantic populations. Diversity drops in marginal northern and in central Mediterranean populations: six groups: N Norway, S Norway-Sweden-Germany, Netherlands, Scotland–Morocco, W Mediterranean, Aegean (see *h* column).	6
Norway lobster (*Nephrops norvegicus*)	5600	12	379	0.933	0.0057	0.016[b]	0.005	–	–	–	4 groups. No IBD nor Atlantic Mediterranean divide. Recent expansion. No Mediterranean glacial refuge.	7
Spiny lobster (*Palinurus gilchristi*)	1100	4	187	0.858	0.0042	0.000	–	–	–	–	Panmixia. Long larval pelagic stage and dispersal in Aghulas Current. Post-glacial expansion over submerged Aghulas Bank..	8
Japanese mitten crab (*Eriocheir japonica*)	4200	19	666	0.711<br>0.341<br>0.121	0.030<br>0.011<br>0.001	0.66–0.92[c]	0.10[c]	–	–	–	3 major groups corresponding to island groups (42.3% of total variation), but less among populations within groups (1.8%).	9

											Some isolation between populations in Japan’s rivers and estuaries. Strong isolation between islands.	
Blue crab (*Callinectes sapidus*)	4600	14	176	0.670<br>0.948<br>0.850<br>0.941<br>0.830	0.010<br>0.022<br>0.017<br>0.020<br>0.021	0.04[b]	–	–	–	–	Estuarine and coastal habitats. Significant differentiation among populations, but no geographic pattern. Lower diversities in north, and possible in ecological marginal population is Keys and Mexico. Deep mtDNA genealogy, but signature of recent expansion.	10
Antarctic krill (*Euphausia superba*)	7500	12	641	0.856	0.0014	-0.0009	-0.0018	2	-0.0008	0.0034	Panmixia. Mobile pelagic adults and larvae lead to high levels of gene flow.	11

Significance: [a] $P < 0.05$, [b] $P < 0.01$, [c] P ¸0.001.

[d]References: 1. This study; 2. Sotelo et al. (2008); 3. Domingues et al. (2010); 4. Roman and Palumbi (2004); 5. Kenchington et al. (2009); 6. Triantafyllidis et al. (2005); 7. Stamatis et al. (2004); 8. Tolley et al. (2005); 9. Yamasaki et al. (2006); 10. McMillen-Jackson and Bert (2004); 11. Bortolotto et al. (2011).

[e]SNP loci.

References

Bortolotto, E., A. Bucklin, M. Mezzavilla, L. Zane, and T. Paternello. 2011. Gone with the currents: lack of genetics differentiation at the circum-continental scale in the Antarctic krill *Euphausia superb*. *BMC Genetics* **12**:32.

Domingues, C. P., S. Creer, M. I. Taylor, H. Queiroga, and G. R. Carvalho. 2010. Genetic structure of *Carcinus maenas* within its native range: larval dispersal and oceanographic variability. *Marine Ecology Progress Series* **410**:111–123.

Kenchington, E. L., G. C. Harding, M. W. Jones, and P. A. Prodöhl. 2009. Pleistocene glaciations events shape genetic structure across the range of the American lobster, *Homarus americanus*. *Molecular Ecology* **18**:1654–1667.

McMillen-Jackson, A. L., and T. M. Bert. 2004. Mitochondrial DNA variation and population genetic structure of the blue crab *Callinectes sapidus* in the eastern United States. *Marine Biology* **145**:769–777.

Roman, J., and S. R. Palumbi. 2004. A global invader at home: population structure of the green crab, *Carcinus maenas*, in Europe. *Molecular Ecology* **13**:2891–2898.

Triantafyllidis, A. P. Apostolidis, V. Katsares, E. Kelly, J. Mercer, M. Hughes, K. E. Jørstad, A. Tsolou, R. Hynes, and C. Triantaphyllidis. 2005. Mitochondrial DNA variation in the European lobster (*Homarus gammarus*) throughout the range. *Marine Biology* **146**:223–235.

Sotelo, G., P. Morán, L. Fernández, and D. Posada. 2008. Genetic variation of the spine spider crab *Maja brachydactyla* in the northeastern Atlantic. *Marine Ecology Progress Series* **362**:211–223.

Stamatis, C., A. Triantafyllidis, K. A. Moutou, and Z. Mamuris. 2004. Mitochondrial DNA variation in Northeast Atlantic and Mediterranean populations of Norway lobster, *Neprops norvegicus*. *Molecular Ecology* **13**:1377–1390.

Tolley, K. A., J. C. Groeneveld, K. Gopal, and C. A. Matthee. 2005. Mitochondrial DNA panmixia in spiny lobster *Palinurus gilchristi* suggests a population expansion. *Marine Ecology Progress Series* **297**:225–231.

Yamasaki, I., G. Yoshizaki, M. Yokoto, C. A. Strüssmann, and S. Watanabe. 2006. Mitochondrial DNA variation and population structure of the Japanese mitten crab *Eriocheir japonica*. *Fisheries Science* **72**:299–309.

Table S7 Summary statistics from mismatch distributions

Mismatch statistics										
			Sudden population expansion				Spatial population expansion			
Sample	Mean	Variance	Tau	Θ_0	Θ_1	*SSD*	Tau	Θ	*M*	*SSD*
1	5.60	11.77	7.58	0.002	13.059	0.0055	4.858	2.467	5.527	0.0070
2	5.70	13.87	8.96	0.00	11.042	0.0118	5.588	3.891	2.607	0.0164
3	5.34	13.35	7.69	0.00	10.410	0.0346	5.602	1.428	3.318	0.0211
4	4.05	14.46	9.12	0.00	4.012	0.0420	5.749	1.427	1.146	0.0198
5	3.44	11.41	No convergence				5.853	1.902	0.725	0.0231
6	3.37	13.49	8.43	0.00	1.807	0.0620	6.511	0.748	0.826	0.0176
7	3.09	13.05	No convergence				5.810	0.448	0.806	0.0332
8	3.56	16.83	No convergence				7.412	0.704	0.729	0.0127
9	3.15	14.22	No convergence				7.010	0.834	0.605	0.0164
10	4.46	12.74	No convergence				6.068	0.914	1.982	0.0323
11	4.20	17.83	8.27	0.00	2.848	0.0772	6.861	0.677	1.089	0.0284
12	0.25	0.25	10.21	0.00	0.287	0.0005	0.227	0.106	3.441	0.0001
13	0.12	0.11	3.00	0.00	0.140	0.0002	0.084	0.106	1.648	0.0001
14	0.36	0.58	3.00	0.00	0.285	0.0021	1.204	0.114	0.305	0.0001
15	0.34	0.26	3.00	0.00	9999	0.0023	0.386	0.006	99999	0.0213
16	0.29	0.27	3.00	0.00	0.375	0.0027	0.313	0.001	99999	0.0002
17	0.29	0.24	3.00	0.00	0.390	0.0040	0.324	0.001	99999	0.0007

Mean = mean number of mismatches; Variance = variance of mismatch mean; SSD = sum of squares of deviation between sudden population expansion model and observed mismatch distribution. Neither SSD nor the raggedness statistic showed significant deviations from the demographic or spatial expansion models. Dates and N_e were not estimated from the mismatch distributions because uncertainties in estimates of a mutation rate produce large uncertainties around the estimates.

The analysis of nucleotide mismatch distributions is often used to estimate historical population parameters when the distribution does not deviated from a unimodal distribution. Tests for a unimodal distribution are not powerful, so many apparently multimodal distributions do not reject the hypothesis of unimodality. Coalescence theory predicts that in an expanding population deep haplotype partitions can also occur that produce multimodal distributions. The crest of fitted model distribution, measured with tau = $2ut$, is taken to represent the time of a population expansion. However, estimates of the mutation rate u are difficult to make (see discussion in Ho *et al*. 2005). In any case, the shape of the mismatch distribution is influenced not only by recent mutations appearing during a population expansion, but also by ancestral polymorphisms in the expanding populations. This produces a bias in the estimate of the 'time-since-expansion' by pushing it into the past.

The following mismatch curves are included in this supplemental section, but were not used to estimate demographic parameters. The use of mismatch estimates to test demographic hypothesis represents an over-interpretation of sequence data in most cases. Hence, parameter estimates and the mismatch distributions for each of the samples (n ranged from 46 to 180) are presented, but without detailed analysis, for those who would like this information. However, in overall appearance, these mismatch distributions are consistent in supporting the hypothesis that western North Pacific populations have deep mtDNA partitions, expected for older populations, and

southeastern populations have very shallow mtDNA haplotype divergences, expected for recently established populations.

Figure S1. Mitochondrial DNA mismatch distributions (bars) of samples of red king crab from the Okhotsk Sea, Norton Sound and Adak Island area. The curves are the expected distributions for a spatial expansion. Distributions and models were estimated with ARLEQUIN.

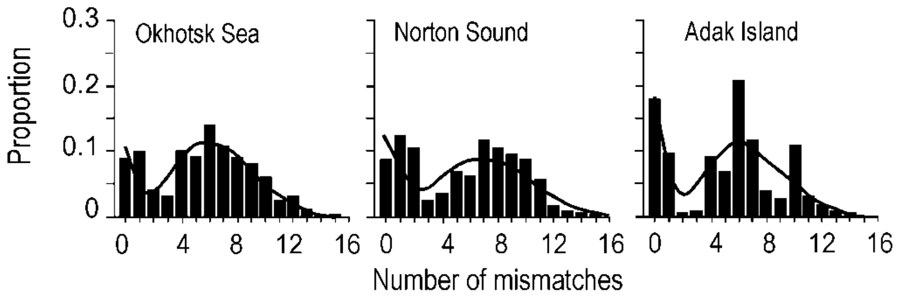

Figure S2. Mitochondrial DNA mismatch distributions in samples of red king crab from the Southeastern Bering Sea and western Gulf of Alaska. The curves are the expected distributions for a spatial expansion. Distributions and models were estimated with ARLEQUIN. Sample locations as in Table 1

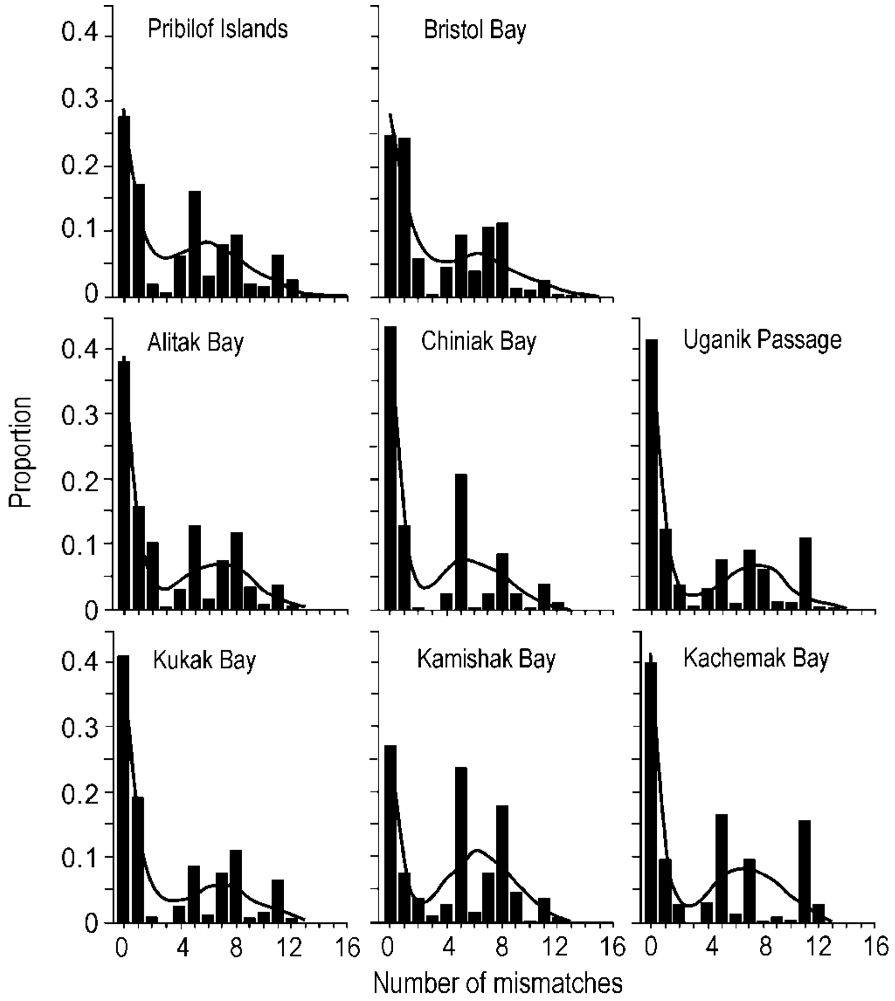

Figure S3. Mitochondrial DNA mismatch distributions of samples of red king crab from Southeast Alaska. Sample locations as in Table 1. The curves are the expected distributions for a spatial expansion. Distributions and models were estimated with ARLEQUIN.

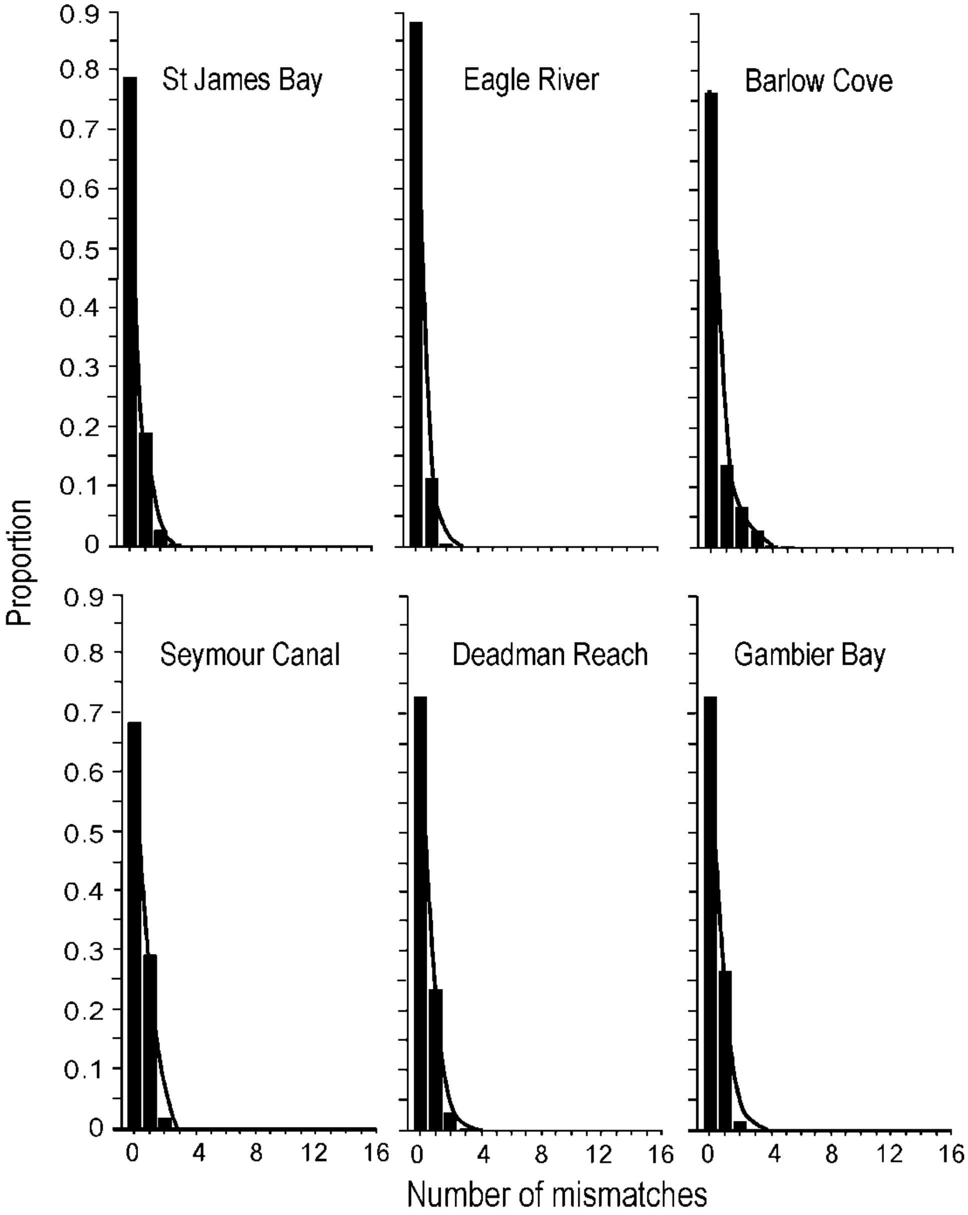

Supplement: Supplementary file 1 [file eva0005-0820-SD1.pdf]
